# Nutritional Evaluation of an EPA-DHA Oil from Transgenic *Camelina sativa* in Feeds for Post-Smolt Atlantic Salmon (*Salmo salar* L.)

**DOI:** 10.1371/journal.pone.0159934

**Published:** 2016-07-25

**Authors:** Mónica B. Betancor, Matthew Sprague, Olga Sayanova, Sarah Usher, Christoforos Metochis, Patrick J. Campbell, Johnathan A. Napier, Douglas R. Tocher

**Affiliations:** 1 Institute of Aquaculture, School of Natural Sciences, University of Stirling, Stirling FK9 4LA, United Kingdom; 2 Department of Biological Chemistry and Crop Protection, Rothamsted Research, Harpenden AL5 2JQ, United Kingdom; 3 Biomar Ltd., North Shore Road, Grangemouth FK3 8UL, United Kingdom; Institut National de la Recherche Agronomique (INRA), FRANCE

## Abstract

Vegetable oils (VO) are possible substitutes for fish oil in aquafeeds but their use is limited by their lack of omega-3 (n-3) long-chain polyunsaturated fatty acids (LC-PUFA). However, oilseed crops can be modified to produce n-3 LC-PUFA such as eicosapentaenoic (EPA) and docosahexaenoic (DHA) acids, representing a potential option to fill the gap between supply and demand of these important nutrients. *Camelina sativa* was metabolically engineered to produce a seed oil with around 15% total n-3 LC-PUFA to potentially substitute for fish oil in salmon feeds. Post-smolt Atlantic salmon (*Salmo salar*) were fed for 11-weeks with one of three experimental diets containing either fish oil (FO), wild-type Camelina oil (WCO) or transgenic Camelina oil (DCO) as added lipid source to evaluate fish performance, nutrient digestibility, tissue n-3 LC-PUFA, and metabolic impact determined by liver transcriptome analysis. The DCO diet did not affect any of the performance or health parameters studied and enhanced apparent digestibility of EPA and DHA compared to the WCO diet. The level of total n-3 LC-PUFA was higher in all the tissues of DCO-fed fish than in WCO-fed fish with levels in liver similar to those in fish fed FO. Endogenous LC-PUFA biosynthetic activity was observed in fish fed both the Camelina oil diets as indicated by the liver transcriptome and levels of intermediate metabolites such as docosapentaenoic acid, with data suggesting that the dietary combination of EPA and DHA inhibited desaturation and elongation activities. Expression of genes involved in phospholipid and triacylglycerol metabolism followed a similar pattern in fish fed DCO and WCO despite the difference in n-3 LC-PUFA contents.

## Introduction

Global demand for omega-3 (n-3) long-chain polyunsaturated fatty acids (LC-PUFA) has significantly increased during the last two decades on the basis of their proven health benefits [1]. In addition world population is expected to increase to 9.1 billion by 2050 [2] increasing pressure on sources of these fatty acids to cover human requirements, estimated to range between 250 mg to 1 g per day of eicosapentaenoic (20:5n-3; EPA) and docosahexaenoic acids (22:6n-3; DHA) combined [3–5]. Therefore, there is mounting concern over the sustainability of sourcing these critical nutrients as they are largely obtained from fish and seafood, which accumulate n-3 LC-PUFA biosynthesised in marine microalgae, the main primary producers [6]. Farmed Atlantic salmon (*Salmo salar*) is an excellent source of n-3 LC-PUFA as this oily fish can accumulate high levels of n-3 LC-PUFA in flesh, with one or two 130g portions being sufficient to supply the amounts of EPA and DHA recommended by EFSA or ISSFAL, respectively [7]. Indeed, aquaculture is the best option to provide these healthy fatty acids as traditional capture fisheries are stagnating with around 53% of marine fish stocks close to being fully exploited, 28% overexploited, 3% depleted and 1% recovering from depletion[8]. However, farmed fish require n-3 LC-PUFA to be included in their diet and this has been achieved by the use of fish oil but, as fish oil is a finite and limited resource, this is an unsustainable practice [9]. Thus, there is a need for alternatives to dietary fish oil that will not only assure good growth and health of the fish, but also maintain the nutritional quality of the final product, particularly high levels of n-3 LC-PUFA.

Currently the predominant substitutes for fish oil are vegetable oils (VO) that can be rich in C_18_ PUFA but naturally lack n-3 LC-PUFA [10]. Dietary VO can generally support normal growth without compromising fish health but, due to the lack of n-3 LC-PUFA, the nutritional quality of the fish fillet is affected, with reduced levels of beneficial EPA and DHA [11–14]. Atlantic salmon have the capability to produce n-3 LC-PUFA from α-linolenic acid (ALA; 18:3n-3) [15] although the capacity is limited to that required by the fish to satisfy its physiological demands [9]. Although research has been performed in order to investigate the “omega-3 trait” in fish, any increased capacity has been insufficient to maintain tissue EPA and DHA in fish fed VO at levels similar to those found in fish fed fish oil [16,17]. Thus, other alternatives to fish oil that are capable of maintaining high n-3 LC-PUFA levels in fish are currently being investigated [9]. Sources such as marine zooplankton (e.g. krill) or phytoplankton (heterotrophic microalgae) represent promising options although biological and technical issues remain that prevent these from being cost-effective alternatives at the moment [9]. Another approach is to develop a new, renewable source of n-3 LC-PUFA, with metabolic engineering of oilseed crops being a viable option. A major advantage of using genetically modified (GM) plants is that oilseed crops already dominate global fat and oil production with well-established infrastructures for the cultivation, harvest, processing, distribution, marketing and utilisation of VO [18]. Production of n-3 LC-PUFA in terrestrial plants has been reported in the oilseed *Camelina sativa* metabolically engineered to contain high-levels of either EPA alone or both EPA and DHA in their seeds [19].

Previously, we investigated the EPA—rich Camelina oil in feeds for post-smolt Atlantic salmon and demonstrated that this novel oil enhanced total n-3 LC-PUFA content in fish fillet compared to fish fed wild-type Camelina oil with no adverse effects on fish growth or health [14,20]. Expression of LC-PUFA biosynthesis genes such as Δ5 and Δ6 desaturases was increased in salmon fed both Camelina oils, although biosynthetic activity was insufficient to maintain flesh DHA levels compared to FO-fed fish. Interestingly, the presence of high levels of EPA in transgenic Camelina did not inhibit/suppress the expression of the biosynthetic genes. In the present study, the efficacy of a second oil derived from transgenic Camelina containing approximately equal proportions of EPA and DHA was evaluated as a replacement for dietary fish oil in feeds for post-smolt Atlantic salmon. The overarching aim was to assess the effects of the novel oil on fish performance and tissue fatty acid composition as well as its impact on metabolism and health.

## Materials and Methods

### Construction of plant expression vector, generation of transgenic plants and extraction of seed oil

A construct containing a cassette of seven genes was used for transformation of *Camelina sativa* [19]. Briefly, the seven-gene construct contained a set of genes optimised for EPA and DHA synthesis: a Δ6-desaturase from *Ostococcus tauri* (OtΔ6), a Δ6 fatty acid elongase from *Physcomitrella patens* (PSE1) a Δ5-desaturase from *Thraustochytrium* sp. (TcΔ5), a Δ12-desaturase from *Phytophthora sojae* (PsΔ12), an ω3-desaturase from *Phytophthora infestans* (Pi- ω3), a Δ5-elongase from *O*. *tauri* and a Δ4-desaturase from *Emiliania huxleyi* as described in detail previously [19]. All genes were individually cloned under the control of seed-specific promoters, and then combined into a single T-DNA transformation vector as described previously [21]. The destination vector contained the dsRed gene with the CsVMV promoter as a visible selection marker via seed coat-specific expression of DsRed. No antibiotic marker was used in the generation of this iteration. All open reading frames for desaturases and elongases were chemically synthesised and codon-optimised for expression in *C*. *sativa*.

Transgenic *C*. *sativa* lines were generated via “floral dip” essentially as described previously [22,23]. Briefly, the designed vector was transferred into *Agrobacterium tumefacians* strain AGL1 and *C*. *sativa* inflorescences immersed in the *Agrobacterium* suspension for 30 sec without application of vacuum. Visual screening for dsRed activity was used to select transgenic seeds expressing the n-3 LC-PUFA biosynthetic pathway. Seeds harvested from transformed plants were illuminated with green LED light and fluorescent seeds identified using a red lens filter. No obvious phenotypic perturbation was observed as a result of modification of the seed oil composition. Full details are in [19]. *C*. *sativa* was grown in a controlled-environment glasshouse at 25°C day/18°C night, 50–60% humidity, and kept under a 16 h photoperiod (long day) at levels of 400 μmol m^-2^ sec^-1^. Oil was extracted from seeds by cold-pressing and solvent extraction (hexane) to maximise yield (PPM, Magdeburg, Germany) with solvent removed by rotary evaporation. The EU authorised anti-oxidant, ethoxyquin was added at 300 ppm to stabilise the final product [24].

### Diets and feeding trial

Three isonitrogenous and isoenergetic diets were formulated to satisfy the nutritional requirements of salmonid fish [25] (Table 1). The diets supplied 45 g/kg crude protein and 26 g/kg crude lipid and were manufactured at BioMar Tech-Centre (Brande, Denmark). The three feeds were produced by vacuum coating identical dry basal extruded pellets with either fish oil (FO), wild-type Camelina oil (WCO) or EPA/DHA-Camelina oil (DCO). Non-defatted fish meal (FM) was employed as the major protein source to ensure EFA requirements were met [25]. Yttrium oxide was added to the experimental diets (0.5 g/kg) as an inert marker for determination of lipid and fatty acid digestibility. A total of 144 post-smolt Atlantic salmon with an average body weight of 256.2 ± 11.7 g (mean ± S.D.) were distributed into 9 seawater tanks (16 per tank) and fed one of the three experimental feeds in triplicate for 11 weeks at the BioMar Research Centre (Hirtshals, Denmark). Prior to the start of the experimental period, during a 1 week acclimation period, fish were fed the WCO diet. The experimental system comprised 1 m^2^, 500 L tanks supplied by flow-through seawater (15 L/min) at ambient temperature that averaged 10.2 ± 0.6°C. Experimental feeds were delivered in excess by belt feeders during 07.00 to 15.30 hrs with uneaten feed collected at the end of the working day in order to accurately determine feed efficiency. All procedures were conducted in accordance with the regulations set forward by the Danish Ministry of Justice and Animal Protection committees by Danish Animal Experiments Inspectorate Permit 2012-15-2934-00573. The experiment was subjected to ethical review by the University of Stirling through the Animal and Welfare and Ethical Review body

**Table 1:** Formulations and proximate compositions of the experimental feeds.

	FO	WCO	DHCO
*Feed ingredients (%)*			
Fish meal	49.8	49.8	49.8
Soy protein concentrate (60%)	12.0	12.0	12.0
Wheat gluten	1.9	1.9	1.9
Wheat	14.0	14.0	14.0
Fish oil	20.8	-	-
Wild-type Camelina oil (Wt-CO)	-	20.8	-
EPA+DHA-Camelina oil (Tr-CO)	-	-	20.8
Vitamins/Minerals	0.8	0.8	0.8
Ytrium oxide	0.05	0.05	0.05
*Analysed composition*			
Dry matter (%)	94.2	94.4	94.4
Protein (%)	45.0	45.5	45.4
Lipid (%)	25.8	25.4	26.2
Ash	8.5	8.8	8.9

### Sample collection

After 10 weeks of feeding, samples of faeces were collected from all the fish from each tank and the faecal samples pooled by tank. Fish were anesthetised with metacaine sulphonate (MS222) (Finquel^®^, Argent Chemical Laboratories, Redmond, WA, USA) 3 hours after their last meal and faecal samples collected from the hind-gut region by gently squeezing the ventral abdominal area [26]. Faecal samples were stored at -20°C prior to lipid and fatty acid analysis. At the end of the trial (11 weeks), blood and tissue was sampled from randomly selected fish. Following 48 h fasting fish were killed by overdose with MS222 and blood from 6 fish per tank collected via the caudal vein by heparinised vacutainers and centrifuged to obtain plasma. Plasma was pooled to obtain three samples per tank and stored at -70°C until further analysis. Nine fish per tank were used for biometric measurements (hepato-somatic and viscera-somatic indices) and tissue analyses. Three whole fish, and samples of muscle (flesh), liver, brain, anterior intestine and pyloric caeca from a further 3 fish per tank were immediately frozen in liquid nitrogen and stored at– 70°C prior to lipid and fatty acid analyses. Additionally, samples of liver from 6 fish per tank were collected, stabilised in RNA Later (Sigma, Poole, UK) and stored at -20°C prior to RNA extraction.

### Biochemical composition

Feeds and whole fish were ground before determination of proximate composition according to standard procedures [27]. Three fish were pooled per tank and three technical replicates for the single batch feeds were analysed. Moisture contents were obtained after drying in an oven at 110°C for 24 h and ash content determined after incineration at 600°C for 16 h. Crude protein content was measured by determining nitrogen content (N x 6.25) using automated Kjeldahl analysis (Tecator Kjeltec Auto 1030 analyzer, Foss, Warrington, UK) and crude lipid content determined gravimetrically after Soxhlet lipid extraction (Tecator Soxtec system 2050 Auto Extraction apparatus).

### Calculations

Feed efficiency and biometric parameters were estimated as follows: Feed intake (FI, g) was estimated by subtracting uneaten feed from fed feed on a dry matter basis. Uneaten feed was recovered daily and corrected for dry matter losses during feeding and collection. Specific growth rate (SGR) = 100 * (lnWo—ln Wf) * D^-1^, where Wo and Wf are the initial and final weights (tanks means), respectively, and D represents the number of feeding days. Feed efficiency ratio (FER) = G * F^-1^, where G is the weight gain and F is the dry matter consumed. Protein efficiency ratio (PER) = weight gain * protein intake. Fulton’s condition factor (k) = 100 * (W/L^3^), where W is the final weight (g) and L is the total length (cm). Liver and empty gastrointestinal tract were used to calculate hepatosomatic index (HSI) = liver weight (g) * 100/Wf (g), and viscerosomatic index (VSI) = viscera weight (g) * 100/Wf (g).

### Digestibility analysis

The apparent digestibility coefficients (ADC) of lipid and selected fatty acids were calculated as: 100 –[100 x (Y_2_O_3_ concentration in feed/Y_2_O_3_ concentration in faeces) x (lipid or fatty acid concentration in faeces/lipid or fatty acid concentration in feed)]. The concentration of individual fatty acids in diets and faeces were calculated based on the relative proportion of each fatty acid compared with a known amount of the internal standard (17:0) added and the total lipid content determined in the samples.

### Tissue and faeces lipid content and fatty acid composition

Samples of flesh, liver, brain, midgut, pyloric caeca from three fish per tank were prepared as pooled homogenates (n = 3 per treatment) whereas faecal samples were analysed on a tank basis (n = 3 per treatment). Total lipid was extracted from approximately 1 g of sample by homogenising in chloroform/methanol (2:1, v/v) using an Ultra-Turrax tissue disrupter (Fisher Scientific, Loughborough, UK), and content determined gravimetrically [28]. Fatty acid methyl esters (FAME) were prepared from total lipid by acid-catalysed transesterification at 50°C for 16 h [29], and FAME extracted and purified as described previously [30]. FAME were separated and quantified by gas-liquid chromatography using a Fisons GC-8160 (Thermo Scientific, Milan, Italy) equipped with a 30 m × 0.32 mm i.d. × 0.25 μm ZB-wax column (Phenomenex, Cheshire, UK), on-column injector and a flame ionisation detector. Data were collected and processed using Chromcard for Windows (version 2.01; Thermoquest Italia S.p.A., Milan, Italy). Individual FAME were identified by comparison to known standards and published data [30].

### Plasma lysozyme activity and myeloperoxidase and protein contents

Plasma lysozyme activity was based on the lysis of lysozyme-sensitive *Microccoccus lysodeikticus* as described by [31]. Total myeloperoxidase (MPO) content in plasma was measured following the procedure described by [32]. Protein content of plasma was determined by the Pierce bicinchoninic acid (BCA) protein assay kit (Thermo Scientific, IL, USA) based on the conversion of Cu^2+^ to Cu^+^ under alkaline conditions (Biuret reaction) using bovine serum albumin (BSA) as a standard.

### RNA extraction and cDNA synthesis

Liver from eighteen individual fish per dietary treatment were homogenized in 1 ml of TriReagent^®^ (Sigma-Aldrich, Dorset, UK), total RNA following manufacturer’s instructions and cDNA synthesized as detailed in [33].

### Microarray hybridisations and image analysis

Transcriptome analysis of liver was performed using an Atlantic salmon custom-made oligoarray with 44k features per array on a four-array-per-slide format (Agilent Technologies UK Ltd., Wokingham, UK), ArrayExpress accession number A-MEXP-2065. A dual-label experimental design was employed for the microarray hybridisations with Cy3-labelled test samples competitively hybridised to a common Cy5-labelled pooled-reference per array. A total of 18 arrays were utilised. The common reference consisted of a pool of equal amounts of amplified RNA from each individual experimental sample.

Sample indirect labelling and hybridization were performed as reported in [14]. Briefly, 250 ng of total RNA were amplified (TargetAmpTM 1-Round Aminoallyl-aRNA Amplification Kit 101. Epicentre, Madison, Wisconsin, USA) and experimental and pooled reference labelled with Cy3 or Cy5 respectively (GE HealthCare, Little Chalfont, UK). Microarray hybridisations were performed in SureHyb hybridisation chambers in a DNA Microarray Hybridisation Oven (Agilent Technologies) with 825 ng of Cy3-labelled experimental biological replicate and Cy5-labelled reference pool being combined and total volume made up to 35 μl with nuclease-free water. Scanning was performed at 5 μm resolution using an Axon GenePix 4200AL Scanner (MDS Analytical Technologies, Wokingham, Berkshire, UK). Laser power was kept constant (80%) and PMT adjusted for each channel such than less than 0.1% features were saturated and that the mean intensity ratio of the Cy3 and Cy5 signals was close to one. Details of the microarray experiment were submitted to ArrayExpress under accession number E-MTAB-4621.

### Quantitative real time PCR

Expression of candidate genes of interest (*fatty acyl desaturases 5* and *6* and *fatty acyl elongases 2*, *5a* and *5b*) was determined by quantitative PCR (qPCR) in liver of fish from all treatments (S1 Table). Results were normalised using reference genes, *elongation factor 1-α* (*ef1a*) and *cofilin-2* (*cfl2*), chosen as the most stable according to GeNorm. Primers were designed using Primer 3 [34] in regions that included the microarray probes. qPCR was performed using a Biometra TOptical Thermocycler (Analytik Jena, Goettingen, Germany) in 96-well plates in duplicate 20 μl reaction volumes containing 10 μl of Luminaris Color HiGreen qPCR Master Mix (Thermo Scientific), 1 μl of the primer corresponding to the analysed gene (10 pmol), 3 μl of molecular biology grade water and 5 μl of cDNA, with the exception of the reference genes, which were determined using 2 μl of cDNA. In addition, amplifications were carried out with a systematic negative control (NTC-no template control) containing no cDNA. Standard amplification parameters contained an UDG pre-treatment at 50°C for 2 min, an initial activation step at 95°C for 10 min, followed by 35 cycles: 15 s at 95°C, 30 s at the annealing Tm and 30 s at 72°C.

### Statistical analysis

All data are means ± S.D. (n = 3) unless otherwise specified. Percentage data were subjected to arcsin square-root transformation prior to statistical analyses. Data were tested for normality and homogeneity of variances with Levene’s test prior to one-way analysis of variance (ANOVA) followed by a Tukey-Kramer HSD multiple comparisons of means. All statistical analyses were performed using SPSS software (IBM SPSS Statistics 19; SPSS Inc., Chicago, IL, USA). Statistical analysis of microarray hybridisation data was performed in GeneSpring GX version 12.6.1 (Agilent Technologies, Wokingham, Berkshire, UK) using a Welch (unpaired unequal variance) t-test, at 0.05 significance. No multiple test correction was employed as previous analyses indicated that they were over-conservative for these nutritional data [16]. Data were submitted to the Kyoto Encyclopedia of Genes and Genomes (KEGG;) [35] for biological function analysis. Gene expression results were analysed using the relative expression software tool (REST 2009; http://www.gene-quantification.info/), which employs a pairwise fixed reallocation randomisation test (10,000 randomizations) with efficiency correction [36] to determine the statistical significance of expression ratios (gene expression fold changes) between two treatments.

## Results

### Fatty acid compositions of the dietary oils

The fatty acid profile of the oil (Tr-CO) extracted from seeds of transgenic *Camelina sativa* showed that this crop effectively accumulated high levels of EPA and DHA (6.0% and 5.1% respectively; Table 2). In addition, the oil also contained appreciable levels of 18:4n-3, 20:4n-3 and docosapentaenoic acid (DPA, 22:5n-3), so that the total n-3 LC-PUFA (20:4n-3, EPA, DPA and DHA) level was almost 15%. None of the aforementioned n-3 PUFA are present in wild-type Camelina oil (Wt-CO) that only contains 18:3n-3 and 20:3n-3 (Table 2), and their presence in Tr-CO was accompanied by decreased proportions of 18:3n-3, 18:1n-9 and 20:1n-9 compared to Wt-CO. The Tr-CO also contained the n-6 LC-PUFA, 20:3n-6 and arachidonic acid (ARA, 20:4n-6), as well as 18:3n-6, whereas both Tr-CO and Wt-CO contained high levels of 18:2n-6. Thus, the Tr-CO displayed a hybrid PUFA composition showing characteristics of FO (LC-PUFA including EPA, DHA and ARA) and VO (18:2n-6) (Table 2).

**Table 2:** Fatty acid composition (percentage of fatty acids) of the oils and feeds.

	Oils			Feeds		
	Fish oil	Wt-CO	Tr-CO	FO	WCO	DCO
14:0	7.9	0.1	n.d.	6.9	0.9	1.0
16:0	20.1	5.2	7.2	19.6	7.5	9.4
18:0	3.9	2.6	5.0	3.8	2.7	4.5
20:0	0.2	1.4	2.8	0.2	1.2	2.0
**Ʃ saturated**^1^	**32.7**	**9.6**	**15.5**	**31.3**	**12.8**	**17.4**
16:1n-7	8.9	0.1	n.d.	8.1	0.9	1.0
18:1n-9	10.8	14.9	7.3	10.9	14.1	8.4
18:1n-7	3.0	0.8	1.6	2.9	1.1	1.6
20:1	2.0	15.0	9.8	2.4	13.1	7.9
22:1	1.8	2.7	1.2	2.8	3.4	2.0
**Ʃ monounsaturated**^2^	**27.2**	**33.9**	**19.9**	**27.8**	**33.2**	**21.4**
18:2n-6	1.2	19.5	20.9	2.6	17.5	19.1
18:3n-6	0.2	n.d.	5.0	0.2	n.d.	4.1
20:2n-6	0.1	2.0	1.3	0.2	1.6	1.0
20:3n-6	0.2	n.d.	1.3	0.2	n.d.	1.0
20:4n-6	1.4	n.d.	2.1	1.3	0.1	1.8
**Ʃ n-6 PUFA**^3^	**3.6**	**21.5**	**30.7**	**4.9**	**19.6**	**27.2**
18:3n-3	0.1	33.6	13.0	0.8	27.6	12.4
18:4n-3	2.1	n.d.	3.9	2.1	0.4	3.8
20:3n-3	0.1	1.4	0.8	0.1	1.1	0.6
20:4n-3	0.6	n.d.	2.3	0.6	0.1	2.0
20:5n-3	17.0	n.d.	6.0	15.4	1.7	6.5
22:5n-3	1.9	n.d.	1.5	1.7	0.2	1.5
22:6n-3	9.5	n.d.	5.1	10.7	3.0	6.9
**Ʃ n-3 PUFA**	**31.3**	**35.0**	**32.7**	**31.4**	**34.1**	**33.7**
**Ʃ PUFA**^4^	40.1	56.5	63.4	**40.9**	**54.0**	**61.2**
**Ʃ n-3 LC-PUFA**	29.0	n.d.	14.9	28.4	5.0	16.9

^1^contains 15:0, 22:0 and 24:0;

^2^contains 16:1n-9 and 24:1n-9;

^3^contains 22:4n-6 and 22:5n-6;

^4^contains C_16_ PUFA.

Fish and FO, fish oil and respective feed; LC- PUFA, long-chain polyunsaturated fatty acid (sum of 20:4n-3, 20:5n-3 22:5n-3 and 22:6n-3); n.d. not detected; Tr-CO and DCO, oil from transgenic Camelina and respective feed; Wt-CO and WCO, oil from wild-type Camelina and respective feed.

### Fish growth performance, feed efficiency and biochemical composition of whole fish

No mortality was observed during the trial (final survival = 100% in all dietary treatments) and fish grew well, more than doubling their initial weight during the experimental period (Table 3). No differences were found between fish fed any of the dietary treatments in any of the fish growth or performance parameters, or indicators of feed efficiency that were evaluated. In contrast to fish performance, differences were found among dietary treatments regarding whole body composition (Table 3). In this regard, the lipid (fat) content of fish fed WCO was higher than that of fish fed the other diets with fish fed DCO showing the lowest lipid content. As expected moisture content was inversely related to lipid content with fish fed WCO having lower water content than fish fed DCO with FO-fed fish displaying intermediate values albeit not significantly different to either WCO or DCO-fed fish.

**Table 3:** Growth performance, survival and basic biometry over the 11-week experimental period.

	FO	WCO	DCO
Initial weight (g)	258.9±7.3	249.1±18.7	260.8±4.9
Final weight (g)	541.6±20.7	543.2±45.1	536.7±28.6
Total length (cm)	35.0±0.5	34.8±0.8	35.0±0.5
FI (g/fish/day)	3.8±0.1	3.9±0.4	3.7±0.3
SGR (%/day)	0.9±0.2	0.9±0.1	0.9±0.1
FER	1.1±0.0	1.2±0.1	1.2±0.0
PER	2.4±0.0	2.4±0.1	2.4±0.1
k	0.6±0.0	0.7±0.1	0.6±0.0
HSI	1.0±0.1	1.1±0.0	1.1±0.0
VSI	9.6±1.0	10.0±0.4	9.2±0.1
*Whole body proximate composition (% wet weight)*			
Moisture (%)	65.0±0.8^ab^	64.3±0.3^b^	66.1±0.7^a^
Protein	17.3±0.5	17.1±0.2	17.3±0.1
Lipid	14.6±0.2^b^	15.6±0.2^a^	13.7±0.5^c^
Ash	2.3±0.3	2.1±0.5	2.0±0.2

Data are means ± SD (n = 3). Different superscript letters within a row denote significant differences among diets as determined by one-way ANOVA with Tukey’s comparison test (p<0.05). FI, feed intake; SGR, specific growth rate; FER, feed efficiency ratio; PER, protein efficiency ratio; k, Fulton’s condition factor; HSI, hepato-somatic index; VSI, viscero-somatic index.

### Lipid and fatty acid digestibilities

**Fig 1:**
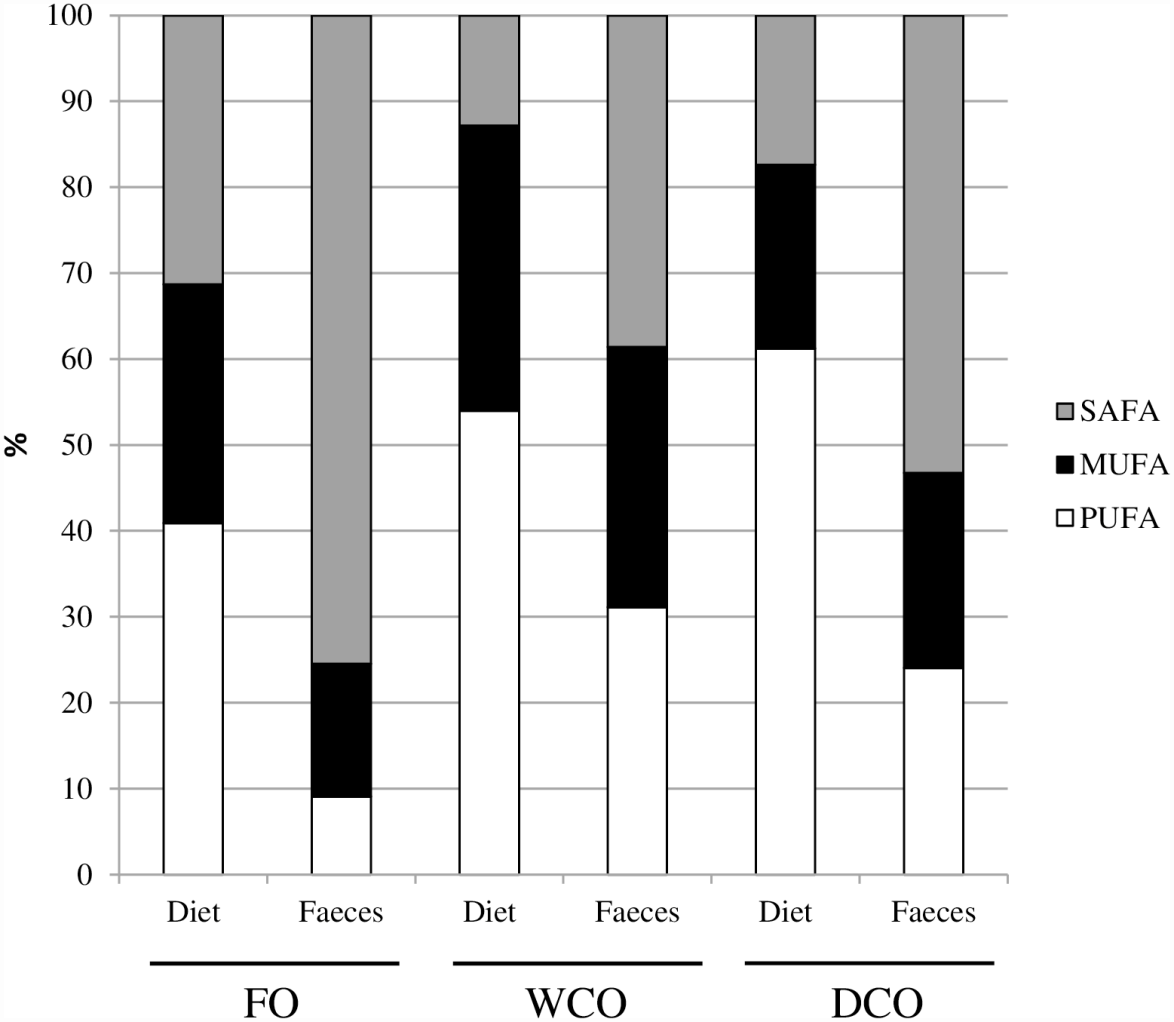
Fatty acid compositions (% of total fatty acids) of the three feeds and faeces of fish fed those feeds. FO, feed containing oil from fish oil; WCO, feed containing oil from wild-type Camelina; DCO; feed containing EPA and DHA.

Oil source affected lipid digestibility with apparent digestibility coefficient (ADC) of lipid being higher in WCO than FO, with DCO showing intermediate values (Table 4). Irrespective of feed, saturated fatty acids were higher and PUFA lower in faeces compared to dietary levels, whereas monoene content was similar in both feed and faeces (Fig 1; S2 Table). High ADCs for EPA and DHA as well as DPA, 18:4n-3, and total n-3 PUFA were observed for FO and DCO. Similarly, ADC for ARA was also higher in FO and DCO than in WCO, but ADCs for other n-6 PUFA did not vary between WCO and DCO feeds. In general, ADCs for saturated and monounsaturated fatty acids were higher for WCO than for FO, with DCO generally intermediate (Table 4). This was clear with ADCs for 14:0, 18:0, 18:1n-9, 20:1n-9, 20:1n-7, 22:1n-11, 22:1n-9 and total monoenes.

**Table 4:** Apparent digestibility coefficient (ADC) of lipid and fatty acids in Atlantic salmon fed the three experimental diets differing in oil source for 11 weeks.

	FO	WCO	DCO
Lipid ADC	93.2 ± 0.8^b^	96.7 ± 0.8^a^	95.2 ± 1.0^ab^
14:0	93.4 ± 0.6^c^	98.0 ± 0.4^a^	96.2 ± 1.2^b^
15:0	90.8 ± 0.8^b^	96.6 ± 0.9^a^	94.3 ± 1.7^a^
16:0	89.1 ± 0.7^b^	96.9 ± 0.9^a^	94.5 ± 1.8^a^
18:0	85.0 ± 1.0^c^	96.0 ± 1.4^a^	91.7 ± 2.5^b^
**Total saturated**^**1**^	89.5 ± 0.7^b^	96.6 ± 1.1^a^	93.2 ± 2.1^b^
16:1n-7	98.6 ± 0.2	98.7 ± 0.3	98.1 ± 0.7
18:1n-9	97.9 ± 0.2^b^	99.1 ± 0.2^a^	98.2 ± 0.7^ab^
18:1n-7	97.3 ± 0.2	98.4 ± 0.3	97.9 ± 0.7
20:1n-9	96.4 ± 0.4^c^	99.0 ± 0.3^a^	98.0 ± 0.7^b^
20:1n-7	94.3 ± 0.4^c^	98.7 ± 0.4^a^	97.3 ± 0.9^b^
22:1n-11	96.3 ± 0.4^b^	98.2 ± 0.5^a^	97.0 ± 1.0^ab^
22:1n-9	92.8 ± 0.7^c^	98.4 ± 0.5^a^	96.6 ± 1.1^b^
**Total monoenes**^**2**^	97.6 ± 0.2^b^	98.9 ± 0.3^a^	97.8 ± 0.8^b^
18:2n-6	96.2 ± 0.4^b^	99.2 ± 0.2^a^	98.9 ± 0.3^a^
20:2n-6	98.0 ± 0.3^b^	99.4 ± 0.1^a^	98.9 ± 0.3^a^
20:4n-6	99.4 ± 0.1^a^	97.5 ± 0.5^b^	99.4 ± 0.2^a^
**Total n-6 PUFA**^**3**^	97.6 ± 0.2^b^	99.2 ± 0.2^a^	99.1 ± 0.3^a^
18:3n-3	98.3 ± 0.1^c^	99.7 ± 0.1^a^	99.4 ± 0.2^b^
18:4n-3	99.6 ± 0.0^ab^	99.4 ± 0.1^b^	99.7 ± 0.1^a^
20:4n-3	99.3 ± 0.0	99.2 ± 0.7	99.5 ± 0.5
20:5n-3	99.6 ± 0.0^a^	98.7 ± 0.2^b^	99.4 ± 0.2^a^
22:5n-3	99.3 ± 0.1^a^	97.8 ± 0.4^b^	99.2 ± 0.2^a^
22:6n-3	98.6 ± 0.1^a^	96.7 ± 0.6^b^	98.1 ± 0.5^a^
**Total n-3 PUFA**	99.2 ± 0.1	99.3 ± 0.1	99.1 ± 0.3
**Total PUFA**^**4**^	99.0 ± 0.1	99.3 ± 0.1	99.1 ± 0.3

Data are means ± SD (n = 3). Different superscript letters within a row denote significant differences among diets as determined by one-way ANOVA with Tukey’s comparison test (p<0.05). FI, feed intake; FCR, feed conversion ratio; SGR, specific growth rate; FER, feed efficiency ratio; PER, protein efficiency ratio; k, Fulton’s condition factor; HSI, hepato-somatic index; VSI, viscero-somatic index.

### Tissue total lipid content

There were no differences in total lipid content of muscle of fish fed the different dietary treatments (Table 5). Similarly, there were no differences in total lipid contents of liver and brain, due to diet (Table 6). However, fish fed DCO showed the lowest total fat contents in pyloric caeca and midgut (p = 0.017 and 0.005 respectively).

**Table 5:** Total lipid content (percentage of the weight) and fatty acid compositions (percentage of total fatty acids) of total lipid of Atlantic salmon muscle (flesh) at the beginning and end of the feeding trial.

	Initial	FO	WCO	DCO
Lipid content	2.3 ± 0.4	3.0 ± 0.7	3.2 ± 0.6	3.4 ± 0.4
14:0	2.3 ± 0.2	4.0 ± 0.3^a^	1.2 ± 0.1^b^	1.2 ± 0.0^b^
16:0	12.9 ± 1.2	16.6 ± 0.1^a^	10.9 ± 0.5^c^	11.7 ± 0.2^b^
18:0	3.2 ± 0.2	3.8 ± 0.1^b^	3.4 ± 0.1^c^	4.4 ± 0.1^a^
**Total saturated**^1^	18.9 ± 1.2	25.0 ± 0.3^a^	16.6 ± 0.4^c^	18.7 ± 0.3^b^
16:1n-7	2.4 ± 0.1	5.1 ± 0.4^a^	1.4 ± 0.1^b^	1.3 ± 0.0^b^
18:1n-9	24.3 ± 2.6	15.9 ± 0.6^b^	18.0 ± 0.8^a^	14.2 ± 0.7^b^
18:1n-7	2.6 ± 0.1	3.1 ± 0.1^a^	1.6 ± 0.1^c^	2.0 ± 0.0^b^
20:1n-11	0.4 ± 0.1	0.3 ± 0.0^a^	0.2 ± 0.0^b^	0.2 ± 0.0^b^
20:1n-9	3.8 ± 0.5	2.4 ± 0.0^c^	8.4 ± 0.6^a^	5.3 ± 0.0^b^
20:1n-7	0.1 ± 0.0	0.2 ± 0.0^b^	0.3 ± 0.0^a^	0.3 ± 0.0^a^
22:1n-11	3.7 ± 0.5	2.3 ± 0.1^a^	1.6 ± 0.3^b^	1.6 ± 0.2^b^
22:1n-9	0.4 ± 0.1	0.3 ± 0.0^c^	1.3 ± 0.1^a^	0.6 ± 0.0^b^
**Total monounsaturated**^2^	38.5 ± 4.0	30.7 ± 1.2^b^	33.7 ± 0.8^a^	26.5 ± 0.8^c^
18:2n-6	12.2 ± 2.3	5.8 ± 0.1^c^	13.4 ± 0.5^b^	15.3 ± 0.3^a^
18:3n-6	0.3 ± 0.1	0.2 ± 0.0^b^	0.3 ± 0.0^b^	1.8 ± 0.1^a^
20:2n-6	0.7 ± 0.1	0.4 ± 0.0^c^	1.8 ± 0.1^a^	1.2 ± 0.0^b^
20:3n-6	0.5 ± 0.1	0.3 ± 0.0^c^	0.6 ± 0.0^b^	1.7 ± 0.1^a^
20:4n-6	0.7 ± 0.2	1.1 ± 0.0^b^	0.5 ± 0.0^c^	1.5 ± 0.1^a^
22:5n-6	0.2 ± 0.0	0.1 ± 0.0^c^	0.2 ± 0.0^b^	0.4 ± 0.0^a^
**Total n-6 PUFA**^**3**^	14.5 ± 2.3	8.3 ± 0.1^c^	16.5 ± 0.5^b^	21.9 ± 0.5^a^
18:3n-3	3.0 ± 0.3	1.6 ± 0.0^c^	14.3 ± 1.4^a^	8.1 ± 0.2^b^
18:4n-3	1.0 ± 0.1	1.2 ± 0.1^c^	1.7 ± 0.1^b^	2.3 ± 0.1^a^
20:3n-3	0.2 ± 0.0	0.1 ± 0.0^c^	1.4 ± 0.1^a^	0.7 ± 0.0^b^
20:4n-3	0.7 ± 0.0	0.9 ± 0.0^c^	1.2 ± 0.0^b^	1.9 ± 0.1^a^
20:5n-3	4.0 ± 1.1	8.0 ± 0.3^a^	2.4 ± 0.1^c^	4.3 ± 0.1^b^
22:5n-3	1.2 ± 0.2	3.2 ± 0.1^a^	0.9 ± 0.1^c^	1.8 ± 0.0^b^
22:6n-3	17.3 ± 4.3	19.0 ± 2.0^a^	11.0 ± 1.0^c^	13.6 ± 0.6^b^
**Total n-3 PUFA**	27.6 ± 5.2	34.5 ± 1.7	33.0 ± 0.8	32.7 ± 0.6
**EPA+DHA**	21.3 ± 5.4	27.0 ± 1.9^a^	13.4 ± 1.1^c^	17.9 ± 0.7^b^
**EPA/DHA**	0.2 ± 0.1	0.4 ± 0.1^a^	0.2 ± 0.0^c^	0.3 ± 0.0^b^
**n-3/n-6**	1.9 ± 0.1	4.2 ± 0.2^a^	2.0 ± 0.1^b^	1.5 ± 0.1^c^
**n-3 LC-PUFA**	23.4 ± 5.7	30.9 ± 1.8^a^	15.5 ± 1.1^c^	21.6 ± 0.7^b^

Data are expressed as means ± SD (n = 3). Different superscript letters within a row denote significant differences among diets at the end of the trial as determined by one-way ANOVA with Tukey’s comparison test (p < 0.005).

^1^Includes 15:0, 20:0, 22:0 and 24:0.

^2^Includes 16:1n-9 and 24:1n-9.

DCO, feed containing EPA+DHA oil from transgenic Camelina; DHA, docosahexaenoic acid (22:6n-3); FO, fish oil feed; n-3 LC-PUFA (sum of 20:4n-3, 20:5n-3, 22:5n-3 and 22:6n-3); PUFA, polyunsaturated fatty acid;

WCO, wild-type Camelina oil feed.

**Table 6:** Total lipid content (percentage of wet weight) and fatty acid compositions (percentage of total fatty acids) of total lipid of liver and brain after feeding the experimental diets.

	FO	WCO	DCO
*Liver*			
Lipid content	3.8 ± 0.2	4.6 ± 0.7	3.3 ± 0.2
**Total saturated**	26.3 ± 0.4^a^	16.3 ± 1.7^c^	23.0 ± 0.6^b^
**Total monounsaturated**	22.7 ± 2.7^b^	32.0 ± 4.0^a^	17.4 ± 0.7^b^
18:2n-6	4.2 ± 0.7^b^	11.3 ± 1.2^a^	9.1 ± 1.1^a^
18:3n-6	0.2 ± 0.1^b^	0.2 ± 0.0^b^	0.5 ± 0.1^a^
20:2n-6	0.5 ± 0.0^c^	2.2 ± 0.2^a^	1.3 ± 0.0^b^
20:3n-6	0.4 ± 0.1^c^	1.4 ± 0.1^b^	2.4 ± 0.1^a^
20:4n-6	3.8 ± 0.4^b^	1.5 ± 0.4^c^	6.1 ± 0.5^a^
22:5n-6	0.4 ± 0.0^a^	0.1 ± 0.0^c^	0.3 ± 0.0^b^
**Total n-6 PUFA**	9.7 ± 2.7^c^	16.7 ± 0.9^b^	19.9 ± 0.9^a^
18:3n-3	1.1 ± 0.2^c^	9.4 ± 1.0^a^	4.0 ± 0.8^b^
18:4n-3	0.4 ± 0.2^b^	1.1 ± 0.2^a^	0.7 ± 0.1^ab^
20:4n-3	0.1 ± 0.0^c^	1.5 ± 0.1^a^	0.6 ± 0.0^b^
20:5n-3	8.6 ± 0.3^a^	4.8 ± 1.0^b^	7.0 ± 0.4^a^
22:5n-3	3.3 ± 0.2^a^	1.2 ± 0.1^c^	1.9 ± 0.0^b^
22:6n-3	26.0 ± 3.4^a^	15.2 ± 3.4^b^	23.8 ± 1.5^a^
**Total n-3 PUFA**	40.9 ± 3.0	35.0 ± 3.2	39.7 ± 0.6
**EPA+DHA**	34.6 ± 3.5^a^	20.0 ± 4.4^b^	30.8 ± 1.9^a^
**EPA/DHA**	0.3 ± 0.0	0.3 ± 0.0	0.3 ± 0.0
**n-3 LC-PUFA**	38.9 ± 3.4^a^	23.0 ± 4.6^b^	34.4 ± 1.9^a^
*Brain*			
Lipid content	8.3 ± 0.8	8.3 ± 0.6	8.0 ± 0.2
**Total saturated**	22.8 ± 0.2	22.3 ± 0.7	22.6 ± 0.3
**Total monounsaturated**	37.5 ± 0.7	37.6 ± 0.8	37.2 ± 1.3
18:2n-6	0.9 ± 0.1^b^	2.0 ± 0.5^a^	1.6 ± 0.2^ab^
18:3n-6	0.0 ± 0.0	0.0 ± 0.0	0.1 ± 0.0
20:2n6	0.1 ± 0.0^c^	0.4 ± 0.0^a^	0.3 ± 0.0^b^
20:3n-6	0.2 ± 0.0^c^	0.3 ± 0.0^b^	0.4 ± 0.0^a^
20:4n-6	1.3 ± 0.0^b^	1.0 ± 0.0^c^	1.6 ± 0.0^a^
22:5n-6	0.1 ± 0.0	0.0 ± 0.0	0.0 ± 0.0
**Total n-6 PUFA**	2.6 ± 0.1^b^	3.7 ± 0.6^a^	4.1 ± 0.3^a^
18:3n-3	0.2 ± 0.0^b^	1.4 ± 0.4^a^	0.6 ± 0.1^b^
18:4n-3	0.1 ± 0.0	0.2 ± 0.1	0.2 ± 0.0
20:4n-3	0.3 ± 0.0^b^	0.5 ± 0.0^a^	0.4 ± 0.0^a^
20:5n-3	5.7 ± 0.1^a^	5.1 ± 0.1^b^	5.1 ± 0.1^b^
22:5n-3	1.8 ± 0.0^a^	1.4 ± 0.1^b^	1.5 ± 0.1^b^
22:6n-3	21.3 ± 0.6	20.5 ± 1.1	20.8 ± 1.2
**Total n-3 PUFA**	29.5 ± 0.6	29.5 ± 0.7	28.9 ± 1.3
**EPA+DHA**	27.0 ± 0.7	25.6 ± 1.2	25.9 ± 1.3
**EPA/DHA**	0.3 ± 0.2	0.2 ± 0.1	0.2 ± 0.1
**n-3 LC-PUFA**	29.1 ± 0.7	27.5 ± 1.3	27.9 ± 1.4

Data are expressed as means ± SD (n = 3). Different superscript letters within a row denote significant differences among diets as determined by one-way ANOVA with Tukey’s comparison test (p < 0.005). DCO, feed containing EPA+DHA oil from transgenic Camelina; DHA, docosahexaenoic acid (22:6n-3); FO, fish oil feed; n-3 LC-PUFA (sum of 20:4n-3, 20:5n-3, 22:5n-3 and 22:6n-3); WCO, wild-type Camelina oil feed.

### Fatty acid composition of muscle (flesh)

The fatty acid composition of salmon muscle (flesh) largely reflected the dietary fatty acid composition. Thus, the percentages of EPA, DHA and DPA were highest in fish fed FO, and lowest in fish fed WCO, with fish fed DCO showing intermediate values (Table 5). The highest levels of 20:4n-3 and 18:4n-3 were found in fish fed DCO, whereas highest levels of 18:3n-3 and 20:3n-3 were found in fish fed WCO. Importantly, total n-3 LC-PUFA and EPA + DHA levels were significantly and substantially increased in flesh of DCO-fed fish compared to WCO-fed fish (Table 5). Overall, however, there were no differences in total n-3 PUFA as the lower proportions of n-3 LC-PUFA found in WCO-fed fish were counteracted by higher levels of 18:3n-3. In contrast, 18:2n-6 and total n-6 PUFA were highest in flesh of fish fed DCO with fish fed FO showing the lowest, other than ARA, which was lowest in fish fed WCO (Table 5). Total saturated fatty acids were highest in flesh of FO-fed fish mainly due to the highest content of 16:0, whereas WCO-fed fish showed the lowest saturates with DCO showing intermediate values (Table 5). Flesh of WCO-fed fish displayed the highest monoene content due to the high percentages of 18:1n-9, 20:1n-9 and 22:1n-9, with DCO-fed fish showing the lowest monoene content.

**Fig 2:**
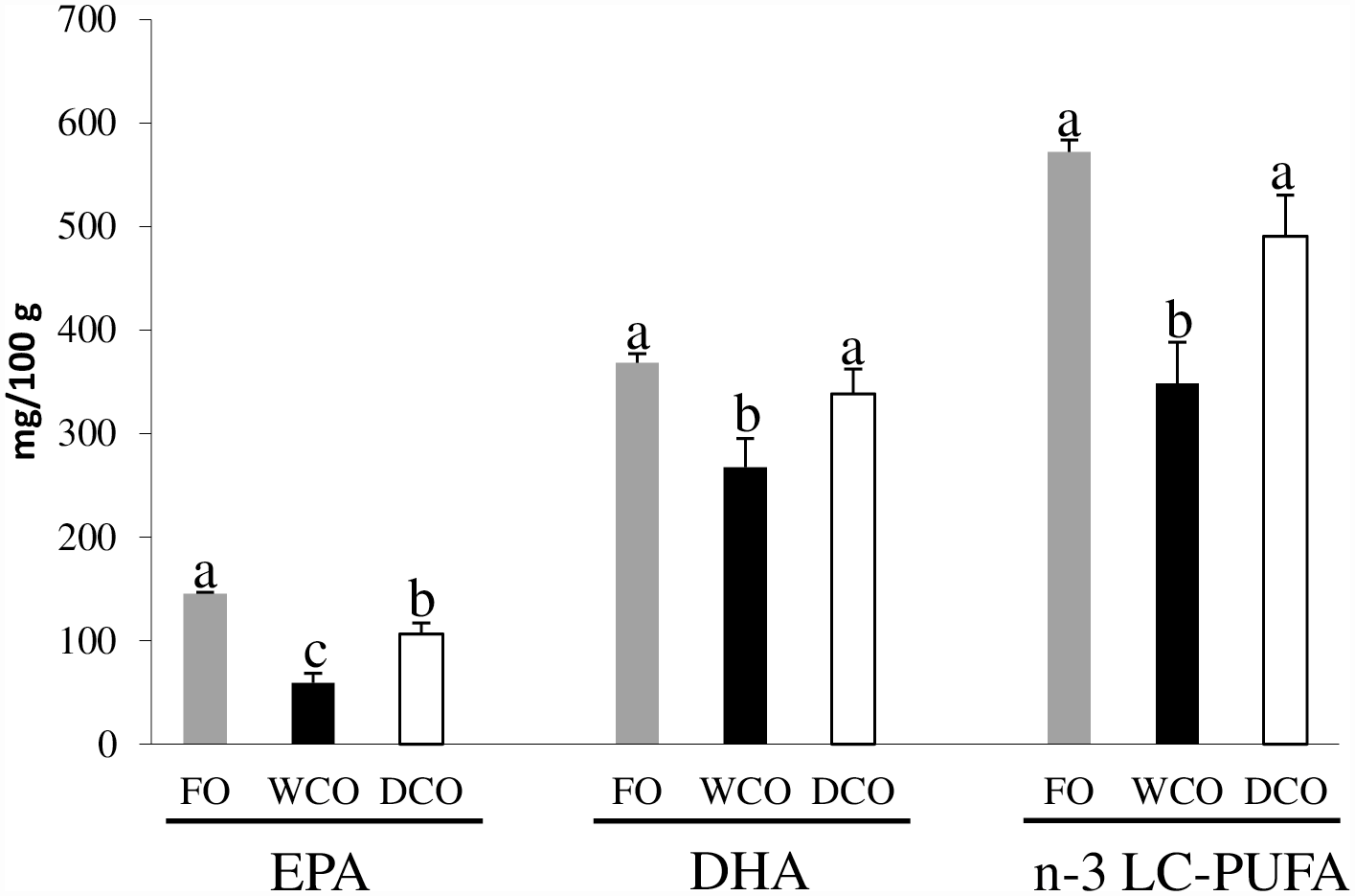
Absolute contents (mg fatty acid/100 g tissue) of EPA, DHA and total n-3 LC-PUFA (EPA+DPA+DHA+20:4n-3) in flesh of Atlantic salmon fed the three experimental diets. FO, feed containing oil from fish oil; WCO, feed containing oil from wild-type Camelina; DCO; feed containing EPA and DHA.

In absolute terms (mg of fatty acid per 100 g of tissue), substantial differences were observed between dietary treatments in EPA, DHA and n-3 LC-PUFA contents of flesh (Fig 2; S3 Table). These data showed that 100 g of flesh from DCO-fed fish delivered almost double the amount of EPA than fish fed WCO with only FO-fed fish providing slightly higher levels (p = 0.003) than DCO. DCO-fed fish flesh also provided higher amounts of DHA than WCO-fed fish (p = 0.017), and similar levels to FO-fed fish (p = 0.282). Total n-3 LC-PUFA (EPA+DHA+DPA+20:4n-3) contents were also similar between muscle of fish fed FO and DCO (p = 0.054), significantly higher than in fish fed WCO (p = 0.001).

### Fatty acid composition of liver and brain

Liver of fish fed DCO displayed similar EPA and DHA levels to fish fed FO, with fish fed WCO displaying the lowest values (Table 6). The percentages of 18:4n-3 and 20:4n-3 were highest in fish fed DCO but n-3 DPA values were intermediate between the levels in fish fed FO and those fed WCO. Although numerically slightly lower, the levels of EPA+DHA and n-3 LC-PUFA in liver of fish fed DCO were not significantly different to those in fish fed FO and significantly higher than in fish fed WCO. Livers of fish fed DCO displayed the highest ARA, 18:3n-6, 20:3n-6 and total n-6 PUFA, with a similar level of 18:2n-6 to that in fish fed WCO (Table 6). Liver in fish fed DCO showed the lowest level of monoenes and an intermediate level of saturates compared to fish fed the other diets.

The fatty acid composition of brain was less affected by diet. There were no effects of diet on total n-3 LC-PUFA, EPA+DHA or DHA levels in brain (Table 6). The proportions of EPA and DPA were slightly lower in brain of fish fed DCO and WCO compared to fish fed FO. Although no differences were observed in 18:4n-3 levels between fish fed the different dietary treatments, higher levels of 20:4n-3 were observed in fish fed both vegetable derived oil treatments (Table 6). Total n-6 PUFA was higher in brain of fish fed WCO and DCO with fish fed DCO having the highest level of ARA (Table 6). Proportions of saturated and monounsaturated fatty acids in brain showed no influence of diet.

### Fatty acid composition of intestinal tissues, pyloric caeca (PC) and mid-gut

Fatty acid compositions of PC and midgut varied between treatments but mainly reflected dietary compositions. In both PC and midgut, highest proportions of EPA were found in fish fed FO, with lowest percentage in fish fed WCO and DCO-fed fish showing an intermediate level (Table 7). The percentages of DHA were similar in PC and midgut of fish fed DCO and FO and higher than that in fish fed WCO, significantly so in the case of PC in fish fed FO. The PC and midgut of fish fed DCO also displayed the highest values for 18:4n-3 and 20:4n-3. Although numerically lower, the variation in EPA+DHA and total n-3 LC-PUFA meant levels in intestinal tissues in fish fed DCO were similar to the levels in fish fed FO, but higher than in fish fed WCO. Total n-6 PUFA were higher in intestinal tissues of fish fed WCO and DCO than in FO-fed fish, with DCO-fed fish having the highest total n-6 PUFA mainly due to the highest levels of 18:2n-6, 18:3n-6, 20:3n-6 and ARA. The PC and midgut in fish fed DCO showed the lowest level of monoenes and intermediate level of saturates compared to fish fed the other diets.

**Table 7:** Total lipid content (percentage of wet weight) and fatty acid compositions (percentage of total fatty acids) of total lipid of pyloric caeca and midgut after feeding the experimental diets.

	FO	WCO	DCO
*Pyloric caeca*			
Lipid content	17.7 ± 4.7^ab^	23.0 ± 1.9^a^	10.3 ± 3.0^b^
**Total saturated**	**23.9 ± 0.5**^**a**^	**15.9 ± 1.8**^**b**^	**19.5 ± 2.9**^**ab**^
**Total monounsaturated**	**38.0 ± 1.3**^**a**^	**39.1 ± 0.7**^**a**^	**23.8 ± 1.6**^**b**^
18:2n-6	8.4 ± 0.6^b^	15.3 ± 1.2^a^	16.9 ± 1.8^a^
18:3n-6	0.3 ± 0.0^b^	0.3 ± 0.0^b^	1.8 ± 0.3^a^
20:2n-6	0.5 ± 0.0^c^	1.8 ± 0.1^a^	1.2 ± 0.1^b^
20:3n-6	0.4 ± 0.0^c^	0.6 ± 0.1^b^	1.7 ± 0.1^a^
20:4n-6	0.9 ± 0.1^b^	0.4 ± 0.2^b^	1.7 ± 0.6^a^
22:5n-6	0.3 ± 0.0^a^	0.1 ± 0.1^b^	0.2 ± 0.1^a^
**Total n-6 PUFA**	**10.8 ± 0.6**^**c**^	**18.1 ± 1.0**^**b**^	**23.8 ± 1.6**^**a**^
18:3n-3	2.0 ± 0.1^c^	14.0 ± 2.4^a^	7.6 ± 1.3^b^
18:4n-3	1.4 ± 0.1^b^	1.7 ± 0.2^ab^	2.2 ± 0.4^a^
20:4n-3	0.9 ± 0.1^b^	1.0 ± 0.1^b^	1.6 ± 0.3^a^
20:5n-3	6.1 ± 1.1^a^	1.4 ± 0.3^c^	3.0 ± 0.2^b^
22:5n-3	2.7 ± 0.2^a^	0.7 ± 0.1^c^	1.4 ± 0.2^b^
22:6n-3	11.7 ± 0.8^a^	6.3 ± 2.0^b^	10.5 ± 2.7^ab^
**Total n-3 PUFA**	24.9 ± 1.6	26.4 ± 0.6	26.9 ± 1.0
**EPA+DHA**	17.9 ± 1.2^a^	7.7 ± 2.3^b^	13.5 ± 2.5^a^
**EPA/DHA**	0.5 ± 0.1^a^	0.2 ± 0.0^b^	0.3 ± 0.1^b^
**n-3 LC-PUFA**	21.4 ± 1.4^a^	9.4 ± 2.4^b^	16.5 ± 2.3^a^
*Midgut*			
Lipid content	10.2 ± 1.4^a^	12.9 ± 2.6^a^	6.5 ± 0.7^b^
**Total saturated**	**24.7 ± 0.6**^**a**^	**18.1 ± 2.1**^**b**^	**21.3 ± 2.5**^**ab**^
**Total monounsaturated**	**35.8 ± 1.1**^**a**^	**36.6 ± 3.1**^**a**^	**28.2 ± 0.7**^**b**^
18:2n-6	7.6 ± 0.7^b^	13.6 ± 1.2^a^	14.8 ± 2.1^a^
18:3n-6	0.2 ± 0.0^b^	0.3 ± 0.0^b^	1.5 ± 0.4^a^
20:2n-6	0.5 ± 0.0^c^	1.6 ± 0.1^a^	1.2 ± 0.1^b^
20:3n-6	0.4 ± 0.0^b^	0.6 ± 0.1^b^	1.6 ± 0.2^a^
20:4n-6	1.3 ± 0.1^ab^	0.7 ± 0.3^b^	2.1 ± 0.5^a^
22:5n-6	0.3 ± 0.1	0.1 ± 0.0	0.3 ± 0.0
**Total n-6 PUFA**	**10.5 ± 0.7**^**c**^	**17.0 ± 0.7**^**b**^	**21.6 ± 2.2**^**a**^
18:3n-3	1.8 ± 0.2^c^	11.7 ± 1.8^a^	6.7 ± 1.5^b^
18:4n-3	1.2 ± 0.0^b^	1.5 ± 0.1^ab^	1.9 ± 0.5^a^
20:4n-3	0.8 ± 0.0^b^	1.0 ± 0.0^ab^	1.5 ± 0.4^a^
20:5n-3	6.0 ± 0.8^a^	1.7 ± 0.3^c^	3.2 ± 0.2^b^
22:5n-3	2.6 ± 0.3^a^	0.8 ± 0.0^c^	1.4 ± 0.1^b^
22:6n-3	14.5 ± 1.0	10.1 ± 3.5	13.3 ± 3.2
**Total n-3 PUFA**	27.1 ± 1.0	27.9 ± 1.8	28.6 ± 0.0
**EPA+DHA**	20.6 ± 1.1	11.8 ± 3.8	18.0 ± 4.0
**EPA/DHA**	0.4 ± 0.1	0.2 ± 0.0	0.3 ± 0.2
**n-3 LC-PUFA**	23.9 ± 1.2^a^	13.6 ± 3.9^b^	19.9 ± 4.5^ab^

Data are expressed as means ± SD (n = 3). Different superscript letters within a row denote significant differences among diets as determined by one-way ANOVA with Tukey’s comparison test (p < 0.005). DCO, feed containing EPA+DHA oil from transgenic Camelina; DHA, docosahexaenoic acid (22:6n-3); FO, fish oil feed; n-3 LC-PUFA (sum of 20:4n-3, 20:5n-3, 22:5n-3 and 22:6n-3); WCO, wild-type Camelina oil feed.

### Plasma lysozyme and myeloperoxidase and protein concentrations

Diet had no significant impact on plasma lysozyme activity, myeloperoxidase (MPO) activity or protein concentration in plasma (Table 8), although WCO-fed fish displayed the lowest MPO activity and plasma protein content (p = 0.376 and 0.085 respectively).

**Table 8:** Lysozyme activity and myeloperoxidase (MPO) and protein contents in plasma of Atlantic salmon fed the three dietary treatments.

	FO	WCO	DCO
Lysozyme activity (units/min/ml)	681.5 ± 56.3	665.8 ± 66.6	660.0 ± 48.3
MPO (Absorbance_450 nm_)	1.90 ± 0.35	1.69 ± 0.27	1.88 ± 0.38
Protein (mg/ml)	87.1 ± 26.5	68.5 ± 24.4	89.8 ± 22.0

Data are expressed as means ± SD (n = 9). DCO, feed containing oil from transgenic Camelina; FO, fish oil feed; MPO, myeloperoxidase activity; WCO, feed containing oil from wild-type Camelina.

### Expression of genes of LC-PUFA biosynthesis in liver

**Fig 3:**
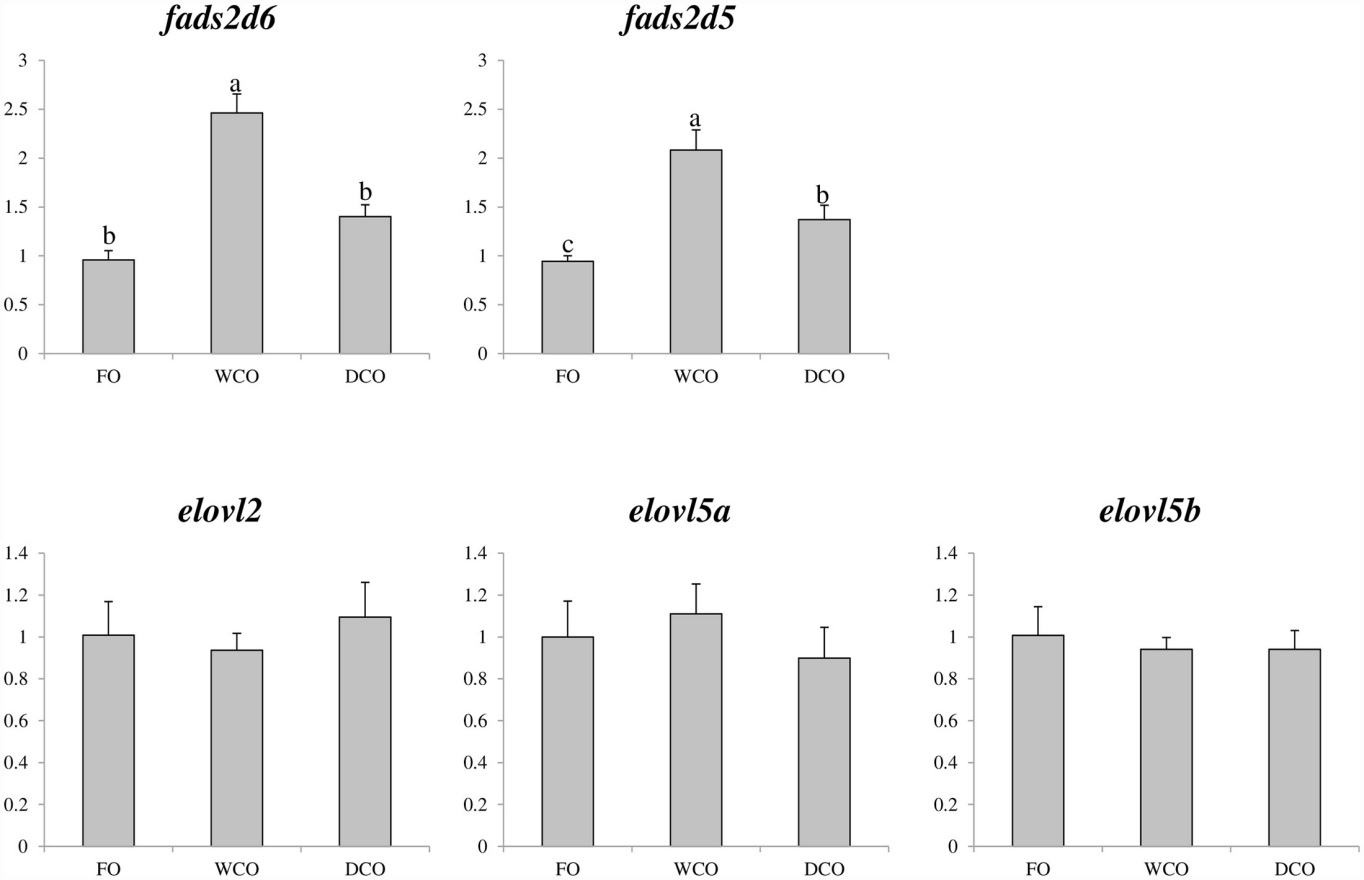
Expression of genes of the LC-PUFA biosynthesis pathway in liver of Atlantic salmon as determined by qPCR. Results are normalised expression ratios (means ± SEM; n = 6). FO, feed containing fish oil as the lipid source; WCO, feed containing wild-type Camelina oil; DCO, feed containing DCO; feed containing EPA and DHA.

Quantification of expression by qPCR of fatty acyl desaturase and elongase genes involved in the biosynthesis of LC-PUFA showed that there was significant up-regulation of *fads2d6* (Δ6 desaturase) and *fads2d5* (Δ5 desaturase) in liver of fish fed WCO compared with fish fed FO and DCO (Fig 3). Expression of *fads2d6* was similar in liver of fish fed DCO and FO, but that of *fads2d5* was higher in fish fed DCO than fish fed FO. There were no significant differences in the expression of the *elovl2*, *elovl5a* or *elovl5b* elongases in liver between fish fed the three dietary treatments (Fig 3).

### Liver transcriptome

**Fig 4:**
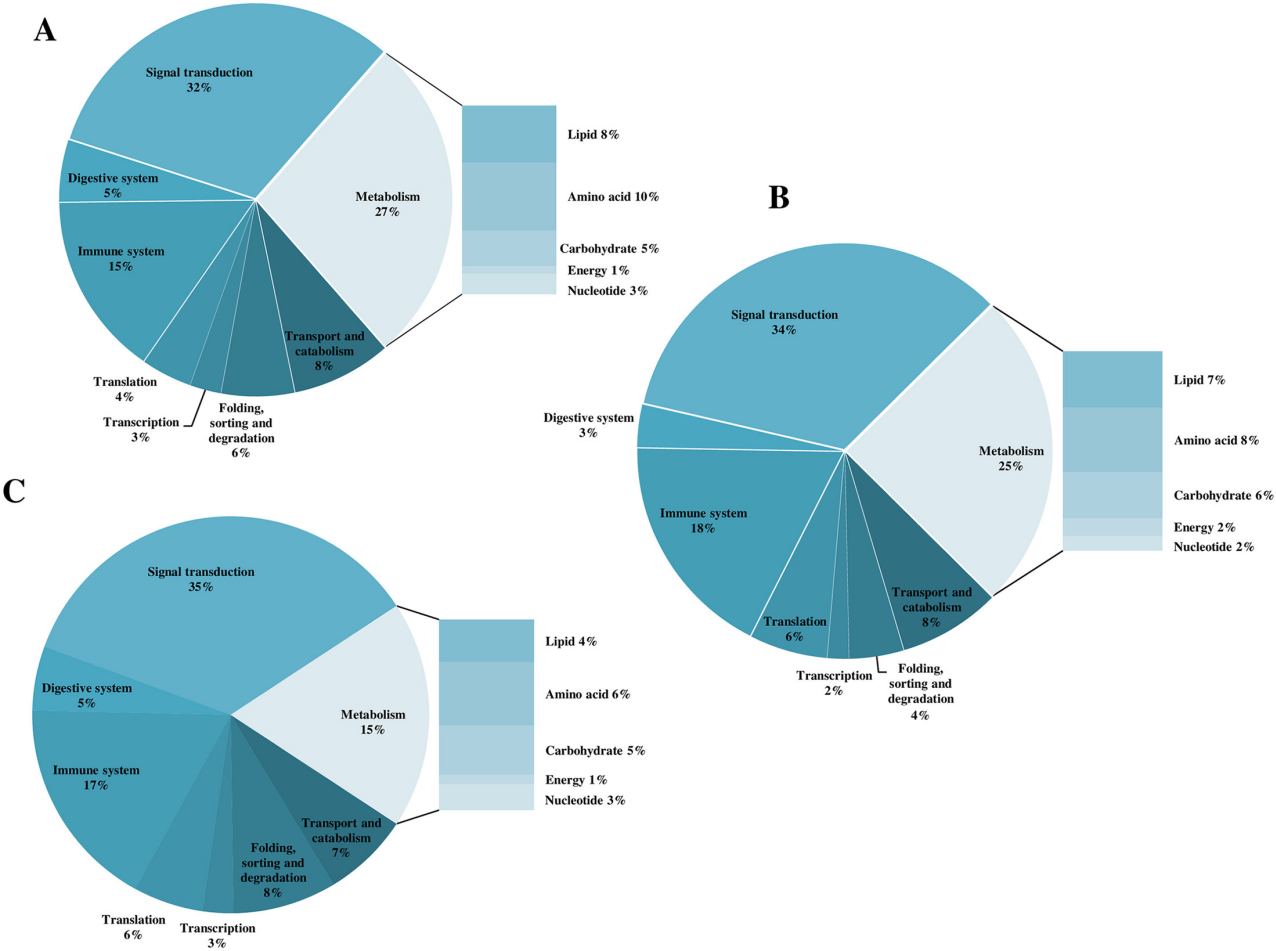
Functional categories of genes differentially expressed in liver of Atlantic salmon post smolts fed diet FO compared to fish fed diet WCO (A) or diet DCO (B), or fish fed DCO compared to those fed WCO (C). Probes with a fold change under 1.3 were removed from the analysis, as well as non-annotated genes and features corresponding to the same gene (Welch t-test, p < 0.005).

Of 43413 specific probes, 26420 passed the quality filtering across the experiment and showed positive and significant signals above background in both channels. Statistical analysis of the microarray data returned a list of 1002 differentially expressed gene (DEG) features in liver between Atlantic salmon fed the DCO and FO diets, compared to 1109 DEG when comparing fish fed the WCO and FO diets, and 944 DEG between fish fed DCO and WCO diets (Table 9). Around 40% of DEG were regulated at a relatively low fold change (FC, 1–1.5) in all contrasts, with the highest percentage of higher FC (> 2.5) found in the DCO/WCO contrast (29.6%; Table 9). DEG with a FC lower than 1.3 were excluded from subsequent analysis, resulting in 931 features for the WCO/FO contrast, 865 for DCO/FO and 794 for DCO/WCO. The DEGs for each comparison were subjected to more detailed analysis by assigning KEGG orthology (KO) numbers and mapping them to a known compendium of metabolic pathways (KEGG). The main pathway categories affected when comparing the livers of WCO-fed fish with those of fish fed either FO and DCO were generally similar being “signal transduction” (32% and 34%, respectively), “metabolism” (27% and 25%, respectively) and “immune system” (15% and 18% respectively) (Fig 4A & 4B). In contrast, the main processes differentially regulated in liver in the comparison between DCO and FO were different, with “signalling” being the main category affected (35%) followed by “immune system” (17%) and “metabolism” (15%) (Fig 4C). Within the “metabolism” category, the representation of each metabolic pathway remained fairly stable among all contrasts, although a reduction in “lipid metabolism” was observed in the contrast DCO/FO (4% compared to 8% and 7% when comparing WCO/FO and DCO/WCO, respectively) (Fig 4).

**Table 9:** Summary of the results of microarray analysis.

	DCO/FO	DCO/WCO	WCO/FO
Total no of probes	44000		
Total no of DEG	1002	944	1109
** Up-regulated genes**	**480**	**441**	**563**
** **FC 1–1.5	178	198	229
** **FC 1.5–2.5	169	132	167
** **FC > 2.5	133	111	167
** Down-regulated genes**	**522**	**503**	**547**
** **FC 1–1.5	196	180	212
** **FC 1.5–2.5	171	155	194
** **FC > 2.5	155	168	140

DCO, feed containing oil from transgenic Camelina; FC; fold change; FO, fish oil feed; WCO, feed containing oil from wild-type Camelina.

**Fig 5:**
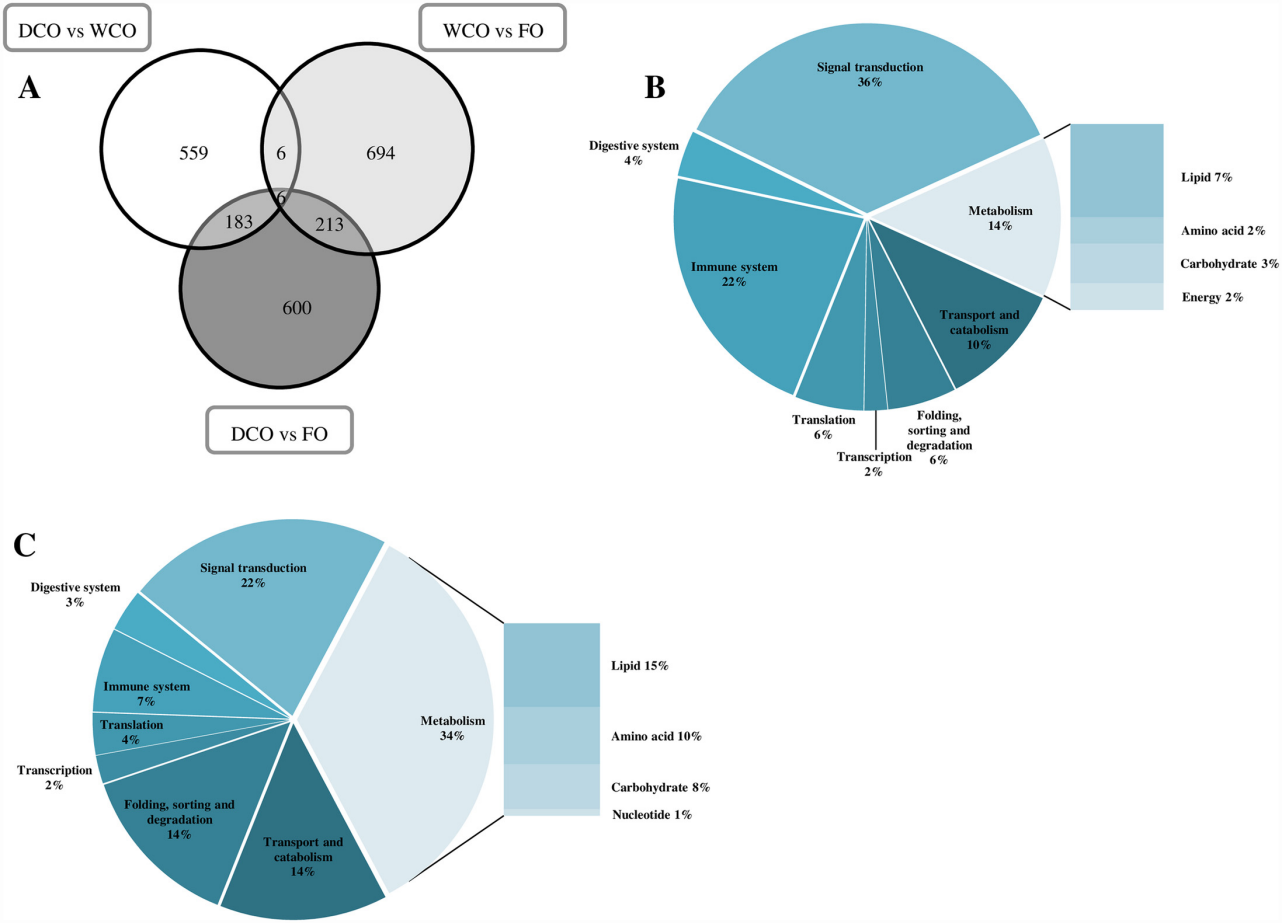
Impact of diet on liver transcriptome of Atlantic salmon fed a diet containing oil from transgenic Camelina (DCO) in comparison with those of fish fed diets containing fish oil (FO) or wild-type Camelina oil (WCO). Venn diagram representing mRNA transcripts differentially expressed in liver of Atlantic salmon fed the three diets with area of the circles scaled to the number of transcripts (Welch t-test, p < 0.05) (A). Distribution by category of common differentially expressed genes in liver of Atlantic salmon fed DCO compared to fish fed FO and WCO (B) or between Atlantic salmon fed FO compared to fish fed WCO and DCO (C).

Of all DEG transcripts, 559 were exclusive to the DCO/WCO comparison, 600 to the DCO/FO, comparison and 189 were DCO-specific (common to both contrasts, p < 0.05; Fig 5A). After removing non-annotated genes and those probes with a FC <1.3, KEGG analysis of the common 189 DEG DCO-specific returned 172 KO terms at p < 0.05 and revealed that the most affected biological categories were “signal transduction” (36%), followed by “immune system” (22%) and “metabolism” (14%). Analysis of the top 100 most significant common 189 DEG according to p value showed increased representation of “signalling” (45.2%) and “metabolism” categories (19%), mainly due to increased lipid, amino acid and energy metabolism features (Fig 5B). All genes followed the same sense of expression in both contrasts (DCO/FO and DCO/WCO), excepting for *long-chain acyl-CoA synthetase*, a gene involved in fatty acid biosynthesis and degradation, which was up-regulated in the first contrast (S4 Table). Two of the other lipid metabolism genes were involved in glycerophospholipid metabolism (*phosphatidate phosphatase* and *phospholipase D3/4*) and bile acid biosynthesis (*cholestanetriol 26 monooxygenase*). Only one gene, belonging to the “immune system” category, was represented by more than one feature (*kindilin 3*) although direction of the regulation diverged between both probes.

Of the 1109 DEG in WCO/FO comparison (Table 9), 219 were commonly regulated in the DCO/FO comparison (Fig 5A), with 186 probes having a FC > 1.3. KEGG analysis of these FO-specific DEG showed that the main category regulated was that of metabolism, accounting for 34%, followed by signalling (22%), with transport and catabolism, and folding, sorting and degradation accounting for 14% each (Fig 5C). When these genes were restricted to the top 100 most significant hits (S5 Table), the category “metabolism” remained at around 32% with “lipid metabolism” still being the main category affected with genes involved in the subcategories, fatty acid synthesis (*delta– 6 fatty acyl desaturase*, *delta– 5 fatty acyl desaturas*e and *palmitoyl—protein thioesterase*, *acetyl-CoA carboxylase*), degradation (*long-chain acyl-CoA synthetase*) and glycerophospholipid metabolism (*glyceronephosphate O-acyltransferase* and *lysophosphatidic acid acyltransferase*). “Folding, sorting and degradation” showed an increased percentage (22%) mainly due to the presence of repeated features for the genes *78kDa glucose-regulated protein*, *DNAJ homolog subfamily C member 3*, *protein disulphide-isomerase A6* and *signal peptidase complex subunit 2*. *Calreticulin*, an endoplasmic reticulum resident protein involved in several processes such as “folding, sorting and degradation”, “transport and catabolism” or “immune system” was up-regulated in liver of FO-fed fish compared to fish fed WCO or DCO.

## Discussion

In the present study a new source of the n-3 LC-PUFA fatty acids, EPA and DHA, from GM *Camelina sativa* was evaluated in Atlantic salmon post-smolts as a potential substitute for fish oil in aquafeeds. The search for alternatives to fish oil in feeds is currently one of the main priorities in the aquafeed industry [37] and this essentially reduces to a search for new sources of EPA and DHA [9], and why this transgenic oilseed crop has been developed [38]. Inclusion of the EPA+DHA Camelina oil in feeds for Atlantic salmon had no detrimental effect on fish growth and performance after 11 weeks of feeding, indicating that this new source of n-3 LC-PUFA could be a potential substitute for dietary fish oil. Results obtained in the present trial are consistent with a previous study employing a GM-derived oil that was high in EPA (~ 20%) where salmon fed this oil as a replacement for fish oil grew well and showed no negative effects on any performance parameter evaluated [14], indicating this oil was also a feasible alternative to fish oil.

Atlantic salmon fed the WCO feed also showed similar growth performance to that obtained in fish fed the diets with higher n-3 LC-PUFA, which was in agreement with previous studies in salmon where good performance with no compromise on health has usually been observed in trials investigating substitutes for dietary fish oil [13–14,16]. This would be influenced by the high inclusion level of fishmeal in the present study that was deliberately chosen to ensure that no dietary treatment would be limited by base pellet composition as the fishmeal is an excellent source of essential amino acids, minerals and vitamins. However, it must be stressed that inclusion of conventional VO, characterized by having high levels of C_18_ fatty acids and devoid of n-3 LC-PUFA, translates into lower contents of EPA and DHA in fish tissues and consequently reduced intake to consumers of farmed fish [7,39,40]. This is a significant issue in aquaculture as fish are not simply an excellent source of protein but also the main source of n-3 LC-PUFA in the human diet with intake associated with beneficial effects on pathologies affecting cardiovascular and neurological systems, and some type of cancers [41–45]. Thus, apart from supporting optimal growth of the fish, the new GM crop-derived oils containing n-3 LC-PUFA enhance the content of these fatty acids in farmed fish compared to feeds containing standard VO.

Lipids are the preferred sources of energy for fish [15] but the differing composition between terrestrial and marine oils can lead to differences in digestibility. For instance, the degree of unsaturation and the chain length of fatty acids can affect their digestibility and, therefore it is important to evaluate the digestibility coefficients of this *de novo* oil. Total lipid ADC was high (> 93%) and higher in the WCO feed compared to the FO feed, in agreement with previous studies in Atlantic salmon [20,46] and other species [47]. Some studies in other teleost species showed lower ADC of lipids in fish fed VO compared with fish fed fish oil, which may be due to the specific VO or formulations used or different digestive capabilities [48–50]. Interestingly, the presence of both EPA and DHA in the DCO feed enhanced the ADC of total fat to a level similar to that found in fish oil, in contrast to that observed with the EPA only Camelina oil tested previously where ADC of total lipid was similar to that of the WCO feed [20]. Higher ADC for LC-PUFA compared to MUFA and SAFA has been reported in several species previously and could explain the difference observed in total fat ADC [12,20,46,47,49,51]. The individual fatty acid ADC were not negatively affected in diet DCO, with enhanced digestibility of LC-PUFA such as ARA, EPA, DPA and DHA compared to diet WCO. The inclusion of DHA in this the present GM oil improved the digestibility of dietary DHA to levels similar to those observed for fish oil compared to the previously (EPA only) GM oil tested making the present oil even more favourable as a fish oil replacement than the former oil [20]. Thus, the EPA+DHA GM Camelina oil can be included in salmon feeds with no apparent negative impact on lipid and fatty acid digestibility, absorption and utilisation compared to fish oil or other VO.

A common side effect of dietary VO, apart from reduced n-3 LC-PUFA levels, has been increased lipid deposition in fish tissues [13,52–54]. Results of the present study supported this, as whole fish lipid content was higher in fish fed WCO than fish fed the FO and DCO diets. However, no differences were observed in flesh lipid content or VSI between fish fed any of the dietary treatments, similar to what was observed with the previous iteration [14]. The mechanism underpinning increased lipid accumulation/deposition is not clear but it has been hypothesised that this could be due to low dietary n-3 LC-PUFA as these fatty acids are known to enhance β-oxidation in Atlantic salmon [55]. Results from the previous high-EPA GM oil showed higher total body fat content in fish fed both wild-type and transgenic Camelina oils compared to fish oil and, thus, we speculated that reduced dietary DHA could be responsible for the increased adiposity as EPA alone did not restore tissue lipid levels to that of fish fed fish oil [14,20]. The present study supports this theory as inclusion of both EPA and DHA in diet DCO reduced total lipid content of salmon. Indeed, body total lipid level was lower in DCO-fed fish than in FO-fed fish. Therefore, the inclusion of the EPA+DHA Camelina oil in the feed led to leaner whole fish without affecting the levels of protein that, in turn, could be another positive effect associated with the use of this new oil.

As mentioned above, one negative effect associated with dietary VO is reduced levels of beneficial n-3 LC-PUFA in fish tissues with accumulation of the shorter C_18_ fatty acids rich in VO [7,15]. The EPA+DHA Camelina oil can be considered a “hybrid” oil as it contains features of both terrestrial and marine oils as the crop has been modified to produce n-3 LC-PUFA from the precursor, 18:3n-3, in the seeds [19]. The DCO feed enhance the deposition of DHA in salmon flesh compared to WCO-fed fish, which contrasted with results obtained with the previous EPA only Camelina oil where DHA levels were similar between fish fed both VO (wild-type and EPA Camelina oils) [14]. However, both GM Camelina oils elevated the proportions of total n-3 LC-PUFA in flesh compared to WCO. This was even more evident when data were presented as absolute values (mg fatty acid/100 g tissue) with flesh of DCO-fed salmon providing the same amount of DHA and total n-3 LC-PUFA as FO-fed fish. In this way, one 140 g portion of DCO-fed Atlantic salmon would supply 623 mg of n-3 LC-PUFA and be sufficient to satisfy daily DHA+EPA requirements recommended by the World Health Organization [56] (250 mg) and International Society for the Study of Fatty Acids and Lipids [3] (500 mg). It must be noted that fish from this trial were not marketable size (0.5 kg) and that fish were fed for a short period of time, thus it is necessary to establish the effect of both oils over longer periods and in market sized fish.

There are further potential health benefits associated with the use of this novel EPA and DHA oil in aquafeeds than just the levels of DHA and n-3 LC-PUFA in flesh. The flesh of fish fed DCO also displayed lower levels of saturated fatty acids and higher levels of PUFA than FO-fed fish. There is evidence that saturates, particularly 12:0, 16:0 and 18:0, raise total cholesterol concentrations and can increase coagulation, inflammation and insulin resistance [57–59]. Dietary guidelines recommend that the daily intake of saturated fatty acids in humans should be limited to 7–10% of energy or less [60]. However, these guidelines have been criticised lately given that the reduction of dietary saturated fat usually results in a diet lower in fat and high in refined carbohydrates, which can cause more undesired health effects than simply eating high saturated fat [61]. Thus, salmon flesh that is naturally rich in protein and low in carbohydrates and contains elevated levels of beneficial PUFA and lower saturated fatty acids as a result of the inclusion of DCO is a food with a desirable ratio of protein:PUFA:saturated fat.

Liver is the main lipid metabolic tissue in fish and is known to be very active in the synthesis of LC-PUFA in salmon [15]. Salmon fed standard VO as a replacement for fish oil show increased expression and activity of LC-PUFA synthesis genes [14,20,62–65]. In the previous study, fish fed both the wild-type Camelina oil and the EPA only Camelina oil displayed increased hepatic expression of *fads2d5*, *fads2d6*, *elovl2*, *elovl5a* and *elovl5b* and the fish fed the EPA Camelina oil also showed active biosynthesis of n-3 LC-PUFA as indicated by increased levels of n-3 DPA and DHA in liver [14]. With the EPA+DHA Camelina oil, the presence of both EPA and DHA resulted in only a mild response in liver gene expression with only *fads2d5* showing increased expression compared to fish fed the FO diet, and there was no accumulation of intermediate metabolites such as n-3 DPA. Thus, despite of having high levels of the substrate for the biosynthesis of n-3 LC-PUFA (18:3n-3), the pathway was suppressed when high levels of the final product (DHA) were also present in the feeds, in contrast to when the dietary oil supplied only EPA. These data confirm an earlier study that suggested the suppressed LC-PUFA biosynthetic activity in salmon fed fish oil was associated with DHA, and that EPA does not inhibit this pathway [66]. Nonetheless, the LC-PUFA biosynthetic capacity of salmonids is limited and thus the inclusion of DCO would probably enhance total n-3 LC-PUFA levels in salmonids compared to the EPA only Camelina oil due to the better retention of DHA despite inhibition of endogenous biosynthesis.

The liver transcriptome analysis of common DEG revealed differences in expression of *fads2d6* and *fads2d5* between fish fed FO and DCO, although it should be noted that desaturases are represented in the microarray by more than 10 features each and only one was detected in this comparison, in contrast to what was found when FO-fed fish were compared to WCO-fed fish (data not shown). It has been demonstrated that discrepancies between microarray and qPCR increase when FC is under 1.4 as was the case in the FO/DCO comparison present study [67]. It was noteworthy that up-regulation of *long-chain acyl-CoA synthetase* (*acsl*) was observed in both WCO and DCO-fed fish when compared to FO-fed fish. After LC-PUFA enter cells, *acsl* converts them to fatty acyl-CoAs that can have numerous metabolic fates, including incorporation into triacylglycerol or phospholipids, or substrates for β-oxidation and protein acylation [68]. However, previous studies in Atlantic salmon did not show differences in the expression of *acsl* in liver between fish fed fish oil and VO, and authors suggested that the function of this gene must not be directly related to β-oxidation, but could be a general fatty acid activator for other lipid metabolism pathways [69]. *Glyceronephosphate O-acyltransferase* (*gnpat*), an enzyme involved in *de novo* biosynthesis and remodelling of glycerophospholipids, was greatly down-regulated in liver of fish fed the WCO and DCO diets compared to those fed FO (10.1- and 5.4-fold in WCO and DCO-fed fish, respectively). Similarly, rats fed rapeseed oil showed decreased activity of mitochondrial GNPAT compared to rats fed fish oil [70]. Thus, despite diet DCO having levels of EPA+DHA more similar to diet FO than WCO, the presence of shorter chain PUFA, typical of terrestrial plants, elicited a response on triacylglycerol and phospholipid metabolism similar to that observed in fish fed other VO. However, another gene involved in glycerophospholipid metabolism, *lysophosphatidic acid acyltransferase*, showed up-regulation in liver of fish fed both Camelina oils, which is in agreement with previous studies in Atlantic salmon, where up-regulation of this pathway was observed in VO-fed fish which also showed higher fat deposition [16]. In addition, the hypotriglyceridemic effect of n-3 LC-PUFA in mammals is widely known and is associated with inhibition of ACSL activity [71] as observed in the present study, which highlights the relevance of this pathway in the increased lipid accumulation found in VO-fed fish. Another gene involved in the synthesis of fatty acids, *acetyl-CoA carboxylase* (*acc*), which provides the malonyl Co-A substrate necessary for the biosynthesis of fatty acids, was up-regulated in fish fed both WCO and DCO, with similar enhanced enzymatic activity found in rainbow trout fed low levels of FO [72]. Similarly, dietary n-3 PUFA suppressed transcription of several lipogenic genes including *acc* in mammals [73].

The liver transcriptome revealed a number of genes that were DCO-specific (genes commonly expressed between the contrasts DCO/FO and DCO/WCO) with the functional category mainly affected being signalling. Liver and pyloric caeca transcriptome of fish fed the EPA only Camelina oil did not show the same pattern, as metabolism was the main category affected followed by translation in liver [14] or signalling in caeca [20]. Immune system was slightly over-represented in DCO-specific DEG in the present study in contrast to the previous study, with three genes related to blood coagulation (*coagulation factor II receptor*, *coagulation factor III* and *component factor D*) showing increased expression in liver of DCO-fed fish. LC-PUFA are precursors for eicosanoid synthesis involved in the control of blood coagulation [74] and blood coagulation can be influenced by diet with fish fed VO showing up-regulation of the pathway [75]. Despite the high representation of the “immune system” category, diet DCO had no effect on the cellular and humoral innate immunity indicators evaluated in the present study. Inclusion of VO did not affect serum lysozyme activity in Atlantic salmon, similar to what has been reported in other teleost species fed a range of different VO [76–80]. Myeloperoxidase (MPO) is an enzyme present in neutrophils with several functions including neutrophil activation [81] and stimulation of macrophages [82], contributing to the general inflammatory response. No effects on plasma MPO values were observed between fish fed the three different dietary treatments suggesting that the number of circulating leucocytes did not vary. In agreement, no significant differences were found in total plasma peroxidases (including MPO) in sea bream (*Sparus aurata*) fed VO [83]. Plasma protein levels can be reduced when plant proteins are used in feeds for fish [84,85], in contrast to what was observed in the present study where there was no variation in this parameter. Thus, fish fed the Camelina oil feeds (WCO and DCO) in the present study did not display any sign of activation or depression of their immune status. However, it is important to stress that many potentially adverse health effects associated with dietary VO are only noticeable when fish are subjected to environmental/pathogen challenges [86]. Thus, it is necessary to also evaluate the response of fish after a stress in order to further assess potential effects of the new GM-oils on fish health.

In summary, the oil containing 6% EPA and 5% DHA, and 15% total n-3 LC-PUFA derived from GM *Camelina sativa* effectively substituted for fish oil in feed for Atlantic salmon, providing sufficient n-3 LC-PUFA to maintain the nutritional quality of the flesh for the human consumer. In addition, the novel oil did not negatively affect the growth performance or overall health of fish. Given the hybrid nature of the oil, with a fatty acid profile between fish oil and VO, additional positive effects to the fillet were observed such as reduced saturated fatty acid content further improving overall health qualities of product. The data showed that the expression of fatty acyl desaturases and elongases in fish fed the DCO diet were similar to those in fish fed the FO diet and lower than fish fed WCO, in contrast to our previous trial where the EPA only Camelina oil induced high expression of these genes indicating that it is DHA that suppresses expression of the genes of the LC-PUFA biosynthetic pathway and not EPA. Further studies are required to investigate the EPA+DHA Camelina oil in large market-size salmon and over the whole production cycle as higher levels of n-3 LC-PUFA could be accumulated in the flesh. The present study demonstrates that genetically modified oilseed crops are a potential solution to fill the gap between demand and supply of EPA and DHA and, specifically, are a viable alternative to fish oil for the supply of n-3 LC-PUFA in aquaculture.

## Supporting Information

S1 Table Primers used for qPCR or PCR analysis. (DOCX)

S2 Table Fatty acid compositions (% of total fatty acids) of total lipid of Atlantic salmon faeces and yttrium contents (g/kg) in faeces and feeds. (DOCX)

S3 Table Fatty acid compositions (mg fatty acid/100 g tissue) of total lipid of Atlantic salmon muscle (flesh) at the end of the feeding trial. (DOCX)

S4 Table Transcripts corresponding to the top 100 most significant features exhibiting differential expression in liver of post-smolt Atlantic salmon fed diet DCO compared to fish fed either diet FO or WCO. Annotated features with a fold change higher than 1.3 (61.8%) are arranged by functional category and within them by increasing p value (assessed by Welch t-test). Numbers in parentheses represents the percentage of genes in each category after removing features belonging to the same gene.
(DOCX)

S5 Table Transcripts corresponding to the top 100 most significant features exhibiting differential expression in liver of post-smolt Atlantic salmon fed diet FO compared to fish fed either the WCO or DCO diets. Annotated features with a fold change higher than 1.3 (61.8%) are arranged by functional category and within them by increasing p value (assessed by Welch t-test). Numbers in parentheses represents the percentage of genes in each category after removing features belonging to the same gene.
(DOCX)
