# Supplementary material for: Nutritional Evaluation of an EPA-DHA Oil from Transgenic Camelina sativa in Feeds for Post-Smolt Atlantic Salmon (Salmo salar L.)
Source: PLoS One. 2016 Jul 25;11(7):e0159934. doi: 10.1371/journal.pone.0159934 (PMC4959691; doi:10.1371/journal.pone.0159934)
Supplement: S1 Table — (DOCX) [file pone.0159934.s001.docx]

**Supplementary Table 1**. Primers used for qPCR or PCR analysis

| Aim | Transcript | Primer sequence (5’→3’) | Amplicon (bp) | Ta | Accession no |
| --- | --- | --- | --- | --- | --- |
| *qPCR* | *fads2d6* | F: TCCTCTGGTGCGTACTTTGT | 163 | 59˚C | NM_001123575.2^a^ |
|  |  | R: AAATCCCGTCCAGAGTCAGG |  |  |  |
|  | *fads2d5* | F: GCCACTGGTTTGTATGGGTG | 148 | 59˚C | NM_001123542.2^a^ |
|  |  | R: TTGAGGTGTCCACTGAACCA |  |  |  |
|  | *elovl2* | F: GGTGCTGTGGTGGTACTACT | 190 | 59˚C | NM_001136553.1^a^ |
|  |  | R: ACTGTTAAGAGTCGGCCCAA |  |  |  |
|  | *elovl5a* | F: TGTTGCTTCATTGAATGGCCA | 150 | 59˚C | GU238431.1^a^ |
|  |  | R: TCCCATCTCTCCTAGCGACA |  |  |  |
|  | *elovl5b* | F: CTGTGCAGTCATTTGGCCAT | 192 | 59˚C | NM_001136552.1^a^ |
|  |  | R: GGTGTCACCCCATTTGCATG |  |  |  |
|  | *cfl2* | F: AGCCTATGACCAACCCACTG | 224 | 60˚C | TC63899^b^ |
|  |  | R: TGTTCACAGCTCGTTTACCG |  |  |  |
|  | *ef1a* | F: CTGCCCCTCCAGGACGTTTACAA | 175 | 60˚C | AF321836^a^ |
|  |  | R: TGTTCACAGCTCGTTTACCG |  |  |  |

^a^ GenBank (<http://www.ncbi.nlm.nih.gov>)

^b^ Atlantic salmon Gene Index (http://compbio.dfci.harvard.edu/tgi)

*fads2d6*, delta-6 fatty acyl desaturase; *fads2d5*, delta-5 fatty acyl desaturase; *elovl2*, fatty acyl elongase 2; *elovl5a*, fatty acyl elongase 5 isoform a; *elovl5b*, fatty acyl elongase isoform b; *cfl2*, cofilin2; *ef1a*, elongation factor 1 alpha.
