# Supplementary material for: Nutritional Evaluation of an EPA-DHA Oil from Transgenic Camelina sativa in Feeds for Post-Smolt Atlantic Salmon (Salmo salar L.)
Source: PLoS One. 2016 Jul 25;11(7):e0159934. doi: 10.1371/journal.pone.0159934 (PMC4959691; doi:10.1371/journal.pone.0159934)
Supplement: S2 Table — (DOCX) [file pone.0159934.s002.docx]

**Supplementary Table 2** Fatty acid compositions (% of total fatty acids) of total lipid of Atlantic salmon faeces and yttrium contents (g/kg) in faeces and feeds.

|  | **FO** | | | **WCO** | | | | **DCO** | | | |
| --- | --- | --- | --- | --- | --- | --- | --- | --- | --- | --- | --- |
| *Faeces* |  |  |  |  |  |  |  |  |  |  |  |
| 14:0 | 10.5 | ± | 0.2 |  | 1.5 | ± | 0.1 |  | 1.6 | ± | 0.0 |
| 16:0 | 49.3 | ± | 0.7 |  | 19.5 | ± | 0.5 |  | 23.4 | ± | 0.2 |
| 18:0 | 13.1 | ± | 0.4 |  | 8.9 | ± | 0.8 |  | 16.9 | ± | 0.3 |
| **Total saturated^1^** | 75.5 | ± | 0.9 |  | 37.5 | ± | 2.1 |  | 54.5 | ± | 0.5 |
| 16:1n-7 | 2.5 | ± | 0.1 |  | 1.0 | ± | 0.1 |  | 0.8 | ± | 0.0 |
| 18:1n-9 | 5.2 | ± | 0.3 |  | 10.3 | ± | 0.4 |  | 6.8 | ± | 0.5 |
| 18:1n-7 | 1.8 | ± | 0.1 |  | 1.4 | ± | 0.1 |  | 1.5 | ± | 0.0 |
| 20:1n-9 | 1.5 | ± | 0.1 |  | 10.6 | ± | 0.3 |  | 6.8 | ± | 0.2 |
| 20:1n-7 | 0.4 | ± | 0.0 |  | 0.4 | ± | 0.0 |  | 0.6 | ± | 0.0 |
| 22:1n-11 | 2.2 | ± | 0.1 |  | 1.6 | ± | 0.0 |  | 1.6 | ± | 0.0 |
| 22:1n-9 | 0.4 | ± | 0.0 |  | 3.1 | ± | 0.2 |  | 1.3 | ± | 0.1 |
| **Total monounsaturated^2^** | 15.4 | ± | 0.7 |  | 30.2 | ± | 0.2 |  | 20.9 | ± | 0.7 |
| 18:2n-6 | 2.3 | ± | 0.0 |  | 12.0 | ± | 0.8 |  | 9.6 | ± | 0.1 |
| 20:2n-6 | 0.1 | ± | 0.0 |  | 0.8 | ± | 0.0 |  | 0.5 | ± | 0.0 |
| 20:3n-6 | 0.1 | ± | 0.0 |  | 0.1 | ± | 0.1 |  | 0.2 | ± | 0.0 |
| 20:4n-6 | 0.2 | ± | 0.0 |  | 0.3 | ± | 0.0 |  | 0.5 | ± | 0.0 |
| **Total n-6 PUFA^3^** | 2.7 | ± | 0.1 |  | 13.3 | ± | 0.8 |  | 11.5 | ± | 0.3 |
| 18:3n-3 | 0.3 | ± | 0.0 |  | 7.7 | ± | 0.4 |  | 3.5 | ± | 0.0 |
| 18:4n-3 | 0.2 | ± | 0.0 |  | 0.2 | ± | 0.0 |  | 0.6 | ± | 0.0 |
| 20:4n-3 | 0.1 | ± | 0.0 |  | 0.1 | ± | 0.1 |  | 0.5 | ± | 0.0 |
| 20:5n-3 | 1.5 | ± | 0.0 |  | 1.8 | ± | 0.2 |  | 1.8 | ± | 0.1 |
| 22:5n-3 | 0.3 | ± | 0.0 |  | 0.4 | ± | 0.0 |  | 0.5 | ± | 0.0 |
| 22:6n-3 | 3.6 | ± | 0.0 |  | 8.5 | ± | 0.8 |  | 5.9 | ± | 0.3 |
| **Total n-3 PUFA** | 6.0 | ± | 0.1 |  | 19.1 | ± | 1.5 |  | 13.1 | ± | 0.4 |
| **Yttrium (g/kg)** | 13.6 | ± | 0.4 |  | 14.2 | ± | 0.8 |  | 14.4 | ± | 0.7 |
|  |  |  |  |  |  |  |  |  |  |  |  |
| *Feeds* |  |  |  |  |  |  |  |  |  |  |  |
| **Yttrium (g/kg)** | 0.3 | | |  | 0.3 | | |  | 0.3 | | |

Data are expressed as means ± SD (n = 3). Different superscript letters within a row denote significant differences among diets as determined by one-way ANOVA with Tukey’s comparison test (p < 0.005). ^1^Includes 15:0, 20:0, 22:0 and 24:0.^2^Includes 16:1n-9 and 24:1n-9. DCO, feed containing EPA+DHA oil from transgenic Camelina; DHA, docosahexaenoic acid (22:6n-3); FO, fish oil feed; PUFA, polyunsaturated fatty acid; WCO, wild-type Camelina oil feed.
