# Supplementary material for: Nutritional Evaluation of an EPA-DHA Oil from Transgenic Camelina sativa in Feeds for Post-Smolt Atlantic Salmon (Salmo salar L.)
Source: PLoS One. 2016 Jul 25;11(7):e0159934. doi: 10.1371/journal.pone.0159934 (PMC4959691; doi:10.1371/journal.pone.0159934)
Supplement: S3 Table — (DOCX) [file pone.0159934.s003.docx]

**Supplementary Table 3** Fatty acid compositions (mg fatty acid/100 g tissue) of total lipid of Atlantic salmon muscle (flesh) at the end of the feeding trial.

|  | **FO** | | | **WCO** | | | | **DCO** | | | |
| --- | --- | --- | --- | --- | --- | --- | --- | --- | --- | --- | --- |
| 14:0 | 85.6 | ± | 27.7^a^ |  | 29.9 | ± | 5.7^b^ |  | 29.8 | ± | 2.8^b^ |
| 16:0 | 355.3 | ± | 87.0 |  | 268.0 | ± | 30.9 |  | 291.3 | ± | 37.0 |
| 18:0 | 81.5 | ± | 22.2 |  | 83.4 | ± | 14.0 |  | 110.8 | ± | 16.0 |
| **Total saturated^1^** | 534.2 | ± | 140.3 |  | 404.2 | ± | 54.6 |  | 464.4 | ± | 61.1 |
| 16:1n-7 | 111.5 | ± | 37.8^a^ |  | 35.1 | ± | 5.8^b^ |  | 33.2 | ± | 3.8^b^ |
| 18:1n-9 | 341.0 | ± | 97.5 |  | 443.4 | ± | 70.6 |  | 355.8 | ± | 44.8 |
| 18:1n-7 | 67.5 | ± | 18.7 |  | 40.1 | ± | 6.4 |  | 49.5 | ± | 5.1 |
| 20:1n-11 | 7.3 | ± | 2.0 |  | 4.8 | ± | 1.3 |  | 5.2 | ± | 0.1 |
| 20:1n-9 | 52.3 | ± | 14.0^c^ |  | 206.3 | ± | 39.2^a^ |  | 132.8 | ± | 16.6^b^ |
| 20:1n-7 | 4.8 | ± | 1.4^b^ |  | 7.2 | ± | 1.3^ab^ |  | 8.0 | ± | 0.7^a^ |
| 22:1n-11 | 49.6 | ± | 13.5 |  | 38.3 | ± | 10.8 |  | 39.8 | ± | 2.9 |
| 22:1n-9 | 6.3 | ± | 1.6^c^ |  | 32.5 | ± | 7.0^a^ |  | 15.7 | ± | 1.9^b^ |
| **Total monounsaturated^2^** | 659.5 | ± | 189.5 |  | 830.1 | ± | 135.7 |  | 661.3 | ± | 75.9 |
| 18:2n-6 | 123.2 | ± | 30.7^b^ |  | 329.6 | ± | 61.7^a^ |  | 383.7 | ± | 54.9^a^ |
| 18:3n-6 | 4.1 | ± | 1.0^b^ |  | 7.0 | ± | 1.4^b^ |  | 44.6 | ± | 7.4^a^ |
| 20:2n-6 | 9.4 | ± | 2.7^c^ |  | 43.1 | ± | 7.0^a^ |  | 29.3 | ± | 4.2^b^ |
| 20:3n-6 | 6.9 | ± | 1.6^b^ |  | 13.8 | ± | 2.2^b^ |  | 42.5 | ± | 6.3^a^ |
| 20:4n-6 | 23.2 | ± | 6.2^b^ |  | 11.1 | ± | 1.3^b^ |  | 38.2 | ± | 5.6^a^ |
| 22:5n-6 | 7.7 | ± | 1.8^a^ |  | 2.8 | ± | 0.5^b^ |  | 5.1 | ± | 0.7^ab^ |
| **Total n-6 PUFA^3^** | 176.9 | ± | 44.8^b^ |  | 407.4 | ± | 73.5^a^ |  | 547.4 | ± | 79.3^a^ |
| 18:3n-3 | 33.1 | ± | 8.1^c^ |  | 353.4 | ± | 73.5^a^ |  | 202.2 | ± | 27.6^b^ |
| 18:4n-3 | 25.1 | ± | 7.8^b^ |  | 42.8 | ± | 8.6^ab^ |  | 57.0 | ± | 10.1^a^ |
| 20:3n-3 | 2.9 | ± | 0.8^c^ |  | 33.7 | ± | 6.2^a^ |  | 17.0 | ± | 2.6^b^ |
| 20:4n-3 | 19.8 | ± | 5.4^b^ |  | 30.3 | ± | 5.3^b^ |  | 48.6 | ± | 5.7^a^ |
| 20:5n-3 | 172.2 | ± | 6.2^a^ |  | 59.4 | ± | 9.0^c^ |  | 106.6 | ± | 10.7^b^ |
| 22:5n-3 | 68.9 | ± | 19.1^a^ |  | 21.5 | ± | 3.4^c^ |  | 45.4 | ± | 5.1^b^ |
| 22:6n-3 | 339.4 | ± | 18.0^a^ |  | 267.6 | ± | 27.7^b^ |  | 338.4 | ± | 24.2^a^ |
| **Total n-3 PUFA** | 730.6 | ± | 64.5 |  | 810.6 | ± | 119.1 |  | 817.2 | ± | 86.1 |
| **EPA+DHA** | 571.5 | ± | 24.0^a^ |  | 326.9 | ± | 36.6^b^ |  | 445.0 | ± | 34.9^ab^ |
| **n-3/n-6** | 4.2 | ± | 0.2^a^ |  | 2.0 | ± | 0.1^b^ |  | 1.05 | ± | 0.1^c^ |
| **n-3 LC-PUFA** | 660.3 | ± | 34.6^a^ |  | 378.7 | ± | 45.1^b^ |  | 539.0 | ± | 45.6^a^ |

Data are expressed as means ± SD (n = 3). Different superscript letters within a row denote significant differences among diets as determined by one-way ANOVA with Tukey’s comparison test (p < 0.005). ^1^Includes 15:0, 20:0, 22:0 and 24:0.^2^Includes 16:1n-9 and 24:1n-9. DCO, feed containing EPA+DHA oil from transgenic Camelina; DHA, docosahexaenoic acid (22:6n-3); FO, fish oil feed; n-3 LC-PUFA (sum of 20:4n-3, 20:5n-3, 22:5n-3 and 22:6n-3); PUFA, polyunsaturated fatty acid;

WCO, wild-type Camelina oil feed.
