# Supplementary material for: Nutritional Evaluation of an EPA-DHA Oil from Transgenic Camelina sativa in Feeds for Post-Smolt Atlantic Salmon (Salmo salar L.)
Source: PLoS One. 2016 Jul 25;11(7):e0159934. doi: 10.1371/journal.pone.0159934 (PMC4959691; doi:10.1371/journal.pone.0159934)
Supplement: S4 Table — Annotated features with a fold change higher than 1.3 (61.8%) are arranged by functional category and within them by increasing p value (assessed by Welch t-test). Numbers in parentheses represents the percentage of genes in each category after removing features belonging to the same gene. (DOCX) [file pone.0159934.s004.docx]

**Supplementary Table 4**. Transcripts corresponding to the top 100 most significant features exhibiting differential expression in liver of post-smolt Atlantic salmon fed diet DCO compared to fish fed either diet FO or WCO. Annotated features with a fold change higher than 1.3 (61.8 %) are arranged by functional category and within them by increasing p value (assessed by Welch t-test). Numbers in parentheses represents the percentage of genes in each category after removing features belonging to the same gene.

| **KO no** | | **DCO/FO** | **DCO/WCO** | **Annotation** |
| --- | --- | --- | --- | --- |
| *Signalling (45.2%)* | | | | |
| K06751 | +2.05 | | +1.90 | Major histocompatibility complex, class I |
| K05872 | +1.40 | | +1.32 | NF-kappa-B inhibitor epsilon |
| K05424 | +3.93 | | +3.92 | Leptin |
| K06062 | +7.91 | | +4.17 | Histone acetyltransferase |
| K04226 | +3.53 | | +3.95 | Arginine vasopressin receptor 1A |
| K04469 | +1.59 | | +1.74 | Nuclear factor of kappa light polypeptide gene enhancer in B cells |
| K03115 | +1.45 | | +1.34 | Casein kinase II subunit beta |
| K03914 | -1.79 | | -1.47 | Coagulation factor II receptor |
| K05636 | +2.54 | | +2.20 | Laminin, beta 1 |
| K04296 | +4.89 | | +6.12 | Leukotriene B4 receptor 1 |
| K05090 | +2.77 | | +2.78 | Macrophage colony-stimulating factor 1 receptor |
| K05031 | +5.59 | | +2.69 | Cystic fibrosis transmembrane conductance regulator |
| K04659 | -1.53 | | -1.51 | Thrombospondin |
| K09291 | +5.82 | | +6.17 | Nucleoprotein TPR |
| K05637 | -2.05 | | -2.47 | Laminin, alpha 1/2 |
| K06752 | +1.32 | | +1.35 | Major histocompatibility complex, class II |
| K05031 | +3.42 | | +3.37 | Cystic fibrosis transmembrane conductance regulator |
| K06089 | -1.40 | | -1.41 | Junctional adhesion molecule 1 |
| K08958 | -3.40 | | -6.64 | Casein kinase I subunit gamma |
|  |  | |  |  |
| *Metabolism (19.0%)* | | | |  |
| *Lipid metabolism (9.5%)* | | |  |  |
| K01897 | +1.50 | | -1.31 | Long-chain acyl-CoA synthetase |
| K15728 | +1.54 | | +1.99 | Phosphatidate phosphatase |
| K00488 | +1.59 | | +1.45 | Cholestanetriol 26-monooxygenase |
| K16860 | -1.54 | | -1.37 | Phospholipase D3/4 |
|  |  | |  |  |
| *Amino acid metabolism (4.8%)* | | |  |  |
| K00799 | +5.79 | | +7.54 | Glutathione S-transferase |
| K00643 | -1.36 | | -1.28 | 5-aminolevulinate synthase |
|  |  | |  |  |
| *Energy metabolism (4.8%)* | | |  |  |
| K01648 | +4.79 | | +5.07 | ATP citrate (pro-S)-lyase |
| K02144 | +1.38 | | +1.45 | V-type H^+^-transporting ATPase subunit H |
|  | | | |  |
| *Immune system (9.5%)* | | |  |  |
| K17084 | -1.32 | | -1.24 | Kindilin 3 |
| K01334 | +1.30 | | +1.30 | Component factor D |
| K17084 | +1.36 | | +1.29 | Kindilin 3 |
| K03901 | +1.64 | | +1.95 | Coagulation factor III |
|  |  | |  |  |
| *Protein folding (4.8%)* | | |  |  |
| K04649 | +1.42 | | +1.37 | Ubiquitin-conjugating enzyme |
| K04440 | +1.73 | | +1.59 | c-Jun N-terminal kinase |
|  |  | |  |  |
| *Cell motility (4.8%)* | | |  |  |
| K05746 | -1.53 | | -1.41 | Enabled |
| K05750 | +3.67 | | +3.45 | NCKJ-associated protein |
|  |  | |  |  |
| *Replication and repair (4.8%)* | | |  |  |
| K10844 | +3.41 | | +3.36 | DNA excision repair protein ERCC-2 |
| K10980 | +3.60 | | +12.03 | Non-homologous end-joining factor 1 |
|  |  | |  |  |
| *Miscellaneous or unknown functions (11.9%)* | | | |  |
| K05641 | +1.60 | | +1.68 | ATP-binding cassette, subfamily A, member 1 |
| K01367 | +2.08 | | +1.33 | Calpain-1 |
| K09562 | -1.68 | | -1.39 | Hsp70 interacting protein |
| K12813 | -1.44 | | -1.48 | Pre-mRNA-splicing factor ATP-dependent RNA helicase DHX16 |
| K09057 | -6.32 | | -3.05 | Hepatic leukemia factor |
