# Supplementary material for: Nutritional Evaluation of an EPA-DHA Oil from Transgenic Camelina sativa in Feeds for Post-Smolt Atlantic Salmon (Salmo salar L.)
Source: PLoS One. 2016 Jul 25;11(7):e0159934. doi: 10.1371/journal.pone.0159934 (PMC4959691; doi:10.1371/journal.pone.0159934)
Supplement: S5 Table — Annotated features with a fold change higher than 1.3 (61.8%) are arranged by functional category and within them by increasing p value (assessed by Welch t-test). Numbers in parentheses represents the percentage of genes in each category after removing features belonging to the same gene. (DOCX) [file pone.0159934.s005.docx]

**Supplementary Table 5**. Transcripts corresponding to the top 100 most significant features exhibiting differential expression in liver of post-smolt Atlantic salmon fed diet FO compared to fish fed either the WCO or DCO diets. Annotated features with a fold change higher than 1.3 (61.8 %) are arranged by functional category and within them by increasing p value (assessed by Welch t-test). Numbers in parentheses represents the percentage of genes in each category after removing features belonging to the same gene

| **KO no** | | | **FO/WCO** | **FO/DCO** | **Annotation** |
| --- | --- | --- | --- | --- | --- |
| *Metabolism (32%)* | | | | | |
| *Lipid (16%)* | | | |  |  |
| K01897 | -1.99 | | | -1.61 | Long-chain acyl-CoA synthetase |
| K01897 | -2.44 | | | -1.50 | Long-chain acyl-CoA synthetase |
| K10226 | -1.82 | | | -1.27 | Delta-6 fatty acyl desaturase |
| K00649 | +10.12 | | | +5.38 | Glyceronephosphate O-acyltransferase |
| K10224 | -1.73 | | | -1.27 | Delta-5 fatty acyl desaturase |
| K13523 | -1.78 | | | -1.56 | Lysophosphatidic acid acyltransferase |
| K01074 | -1.32 | | | -1.27 | Palmitoyl-protein thioesterase |
| K11262 | -2.03 | | | -1.66 | Acetyl-CoA carboxylase |
|  |  | | |  |  |
| *Glycan metabolism (8%)* | | | |  |  |
| K03847 | +1.37 | | | +1.32 | Alpha-1,6-mannosyltransferase |
| K03847 | +1.40 | | | +1.28 | Alpha-1,6-mannosyltransferase |
| K12667 | +4.38 | | | +3.41 | Oligosaccharyltransferase complex subunit delta |
| K07970 | -1.89 | | | -2.11 | Beta-1,3-N-acetylglucosaminyltransferase 3 |
|  |  | | |  |  |
| *Amino acid metabolism (4%)* | | | |  |  |
| K07410 | | -2.06 | | -1.71 | Cytochrome P450, family 1 |
| K00933 | | -1.72 | | -1.82 | Creatine kinase |
|  | |  | |  |  |
| *Carbohydrate metabolism (2%)* | | | |  |  |
| K01837 | +2.09 | | | +1.57 | Bisphosphoglycerate mutase putative |
|  | |  | |  |  |
| *Cofactors and vitamins (2%)* | | | |  |  |
| K10524 | | -1.33 | | -1.26 | Nicotinate riboside kinase |
|  | |  | |  |  |
| *Folding, sorting and degradation (22%)* | | | | |  |
| K09490 | | +1.82 | | +1.76 | 78 kDa glucose-regulated protein |
| K09490 | | +1.70 | | +1.80 | 78 kDa glucose-regulated protein |
| K09490 | | +1.65 | | +1.73 | 78 kDa glucose-regulated protein |
| K09523 | | +1.77 | | +1.60 | DNAJ homolog subfamily C member 3 |
| K09584 | | +1.36 | | +1.43 | Protein disulphide-isomerase A6 |
| K12947 | | +1.40 | | +1.32 | Signal peptidase complex subunit 2 |
| K09584 | | +1.58 | | +1.43 | Protein disulphide-isomerase A6 |
| K12947 | | +1.76 | | +1.39 | Signal peptidase complex subunit 2 |
| K08489 | | +1.54 | | +1.52 | Syntaxin 16 |
| K09517 | | +1.63 | | +1.86 | DNAJ homolog subfamily C member 3 |
| K09523 | | +2.08 | | +1.83 | DNAJ homolog subfamily C member 3 |
| K12305 | | -1.80 | | -1.87 | Ectonucleoside triphosphate diphosphohydrolase 4 |
|  | |  | |  |  |
| *Transport and catabolism (12%)* | | | | |  |
| K15121 | | +2.43 | | +2.29 | Solute carrier family member 44 |
| K13780 | | +1.60 | | +1.58 | Solute carrier family 7 |
| K06618 | | -1.63 | | -1.37 | Retinoblastoma-associated protein |
| K06751 | | +1.53 | | +1.35 | Major histocompatibility complex, class I |
| K17917 | | +1.80 | | +1.46 | Sorting nexin1/2 |
| K05641 | | +1.42 | | +1.22 | ATP-binding cassette, subfamily a, member 1 |
|  | |  | |  |  |
| *Signalling (12%)* | | | |  |  |
| K09028 | | -1.95 | | -1.96 | Transcription factor jun-B |
| K04496 | | +7.66 | | +5.77 | C-terminal binding protein |
| K04288 | | +7.55 | | +5.68 | Sphingosine 1-phosphate receptor 1 |
| K04423 | | -4.17 | | +2.09 | Mitogen-activated protein kinase 12 |
| K04659 | | +1.58 | | +1.38 | Thrombospondin 2/3/4/5 |
| K09028 | | -1.99 | | -2.29 | Transcription factor jun-B |
|  | |  | |  |  |
| *Transcription (4%)* | | | |  |  |
| K12860 | | +2.32 | | +1.97 | Pre-mRNA splicing factors CDC5/CEF1 |
| K12887 | | +4.41 | | +5.82 | Heterogeneous nuclear ribonucleoprotein M |
|  | |  | |  |  |
| *Immune system (2%)* | | | |  |  |
| K07366 | | +6.45 | | +4.35 | GRB2-related adaptor protein 2 |
|  | |  | |  |  |
| *Miscellaneous or unknown functions (6%)* | | | | | |
| K04994 | | | -7.73 | -4.97 | Mucolipin 3 |
| K13826 | | | -1.47 | -1.58 | Hemoglobin subunit alpha |
| K00280 | | | -2.30 | -2.44 | Lysyl oxydase-like 3 |
| K10990 | | | +4.02 | +2.65 | RecQ-mediated genome instability protein 1 |
| K08507 | | | +1.57 | +1.56 | Calreticulin |
| K08507 | | | +1.48 | +1.48 | Calreticulin |
| K08507 | | | +1.35 | +1.31 | Calreticulin |
| K08507 | | | +1.40 | +1.42 | Calreticulin |
